# Long-Read-Resolved, Ecosystem-Wide Exploration of Nucleotide and Structural Microdiversity of Lake Bacterioplankton Genomes

**DOI:** 10.1128/msystems.00433-22

**Published:** 2022-08-08

**Authors:** Yusuke Okazaki, Shin-ichi Nakano, Atsushi Toyoda, Hideyuki Tamaki

**Affiliations:** a Institute for Chemical Research, Kyoto University, Uji, Kyoto, Japan; b Bioproduction Research Institute, National Institute of Advanced Industrial Science and Technology, Tsukuba, Ibaraki, Japan; c Center for Ecological Research, Kyoto University, Otsu, Shiga, Japan; d Advanced Genomics Center, National Institute of Genetics, Mishima City, Shizuoka, Japan; MIT

**Keywords:** freshwater microbial ecosystem, long-read sequencing, metagenome, microdiversity

## Abstract

Reconstruction of metagenome-assembled genomes (MAGs) has become a fundamental approach in microbial ecology. However, a MAG is hardly complete and overlooks genomic microdiversity because metagenomic assembly fails to resolve microvariants among closely related genotypes. Aiming at understanding the universal factors that drive or constrain prokaryotic genome diversification, we performed an ecosystem-wide high-resolution metagenomic exploration of microdiversity by combining spatiotemporal (2 depths × 12 months) sampling from a pelagic freshwater system, high-quality MAG reconstruction using long- and short-read metagenomic sequences, and profiling of single nucleotide variants (SNVs) and structural variants (SVs) through mapping of short and long reads to the MAGs, respectively. We reconstructed 575 MAGs, including 29 circular assemblies, providing high-quality reference genomes of freshwater bacterioplankton. Read mapping against these MAGs identified 100 to 101,781 SNVs/Mb and 0 to 305 insertions, 0 to 467 deletions, 0 to 41 duplications, and 0 to 6 inversions for each MAG. Nonsynonymous SNVs were accumulated in genes potentially involved in cell surface structural modification to evade phage recognition. Most (80.2%) deletions overlapped with a gene coding region, and genes of prokaryotic defense systems were most frequently (>8% of the genes) overlapped with a deletion. Some such deletions exhibited a monthly shift in their allele frequency, suggesting a rapid turnover of genotypes in response to phage predation. MAGs with extremely low microdiversity were either rare or opportunistic bloomers, suggesting that population persistency is key to their genomic diversification. The results concluded that prokaryotic genomic diversification is driven primarily by viral load and constrained by a population bottleneck.

**IMPORTANCE** Identifying intraspecies genomic diversity (microdiversity) is crucial to understanding microbial ecology and evolution. However, microdiversity among environmental assemblages is not well investigated, because most microbes are difficult to culture. In this study, we performed cultivation-independent exploration of bacterial genomic microdiversity in a lake ecosystem using a combination of short- and long-read metagenomic analyses. The results revealed the broad spectrum of genomic microdiversity among the diverse bacterial species in the ecosystem, which has been overlooked by conventional approaches. Our ecosystem-wide exploration further allowed comparative analysis among the genomes and genes and revealed factors behind microbial genomic diversification, namely, that diversification is driven primarily by resistance against viral infection and constrained by the population size.

## INTRODUCTION

In microbial ecology, reconstruction of metagenome-assembled genomes (MAGs) from an uncultured microbial assemblage has become a routine technique that has reshaped and substantially expanded our understanding of prokaryotic diversity (1, 2). However, MAGs are hardly complete (i.e., circularly assembled) due to difficulties in assembling repetitive (e.g., rRNA genes) and hypervariable (microdiverse) regions in a genome coexisting in the same sample (3, 4). In particular, genomic microdiversity hampers metagenomic assembly and results in incompleteness or the absence of a MAG even at deep sequencing depths, which has been recognized as “the great metagenomics anomaly” (5). Moreover, a metagenomic assembler generally tries to generate a consensus long contig rather than fragmented assemblies reflecting different microvariants (3, 6). Consequently, in a metagenomic assembly, genomic microdiversity is either unassembled or masked by a consensus sequence.

Genomic microdiversity provides information essential to understanding microbial ecology and evolution. The hypervariability of genes involved in cell surface structural modification is thought to be a consequence of the virus-host arms race (7, 8). Intraspecies flexibility of the genes responsible for the availability of substrates and nutrients suggests that functionally diversified populations collectively occupy the diverse microniches (9). The degree of genomic microdiversification varies among lineages and is thought to depend on a number of ecological and evolutionary factors, such as mutation rate, generation time, population size, genetic mobility, fitness, and drift (10, 11). However, due to the aforementioned difficulties, a comprehensive investigation of genomic microdiversity covering a consortium of microbes in an ecosystem is challenging, and the universal factors that drive or constrain their genomic diversification remain to be elucidated.

To address this, the present study took a three-step approach. The first was comprehensive metagenomic sampling in an ecosystem. We targeted freshwater bacterioplankton assemblages sampled spatiotemporally (2 depths × 12 months) at a pelagic station on Lake Biwa, a monomictic lake with an oxygenated hypolimnion that harbors one of the best-studied freshwater microbial ecosystems (12–16). The second step was long-read metagenomic assembly, which can overcome the problem of fragmented assembly by using reads longer than a repeat or hypervariable region (17–20). This was done to generate high-quality reference MAGs covering the diversity of bacterioplankton in the lake. The third step was short- and long-read metagenomic read mapping to the MAGs, in which genomic microvariants were identified as inconsistencies between a consensus assembly and mapped reads (21–23). Notably, we aimed to detect two different types of microvariants, single nucleotide variants (SNVs) and structural variants (SVs), namely, insertion, deletion, duplication, or inversion of a genomic sequence. While short-read mapping efficiently detects SNVs due to its high base accuracy (24, 25), it cannot resolve most SVs that are longer than the canonical short-read length (i.e., 150 to 250 bp). SVs are often associated with gains and losses of genes, which account for a large part of genomic and functional heterogeneity among closely related genotypes (9, 10). Here, the limitation of short-read mapping is complemented by long-read mapping, in which SVs can be located with reads discontinuously aligned to a consensus assembly (26–28). Our three-step approach allowed a high-resolution, ecosystem-wide exploration of SNVs and SVs covering the broad spectrum of prokaryotic diversity in the lake. The results were comparatively analyzed from spatiotemporal, phylogenetic, and gene functionality perspectives, aiming at characterizing factors behind the genomic microdiversification.

## RESULTS

### General characteristics of the rMAGs.

**FIG 1:**
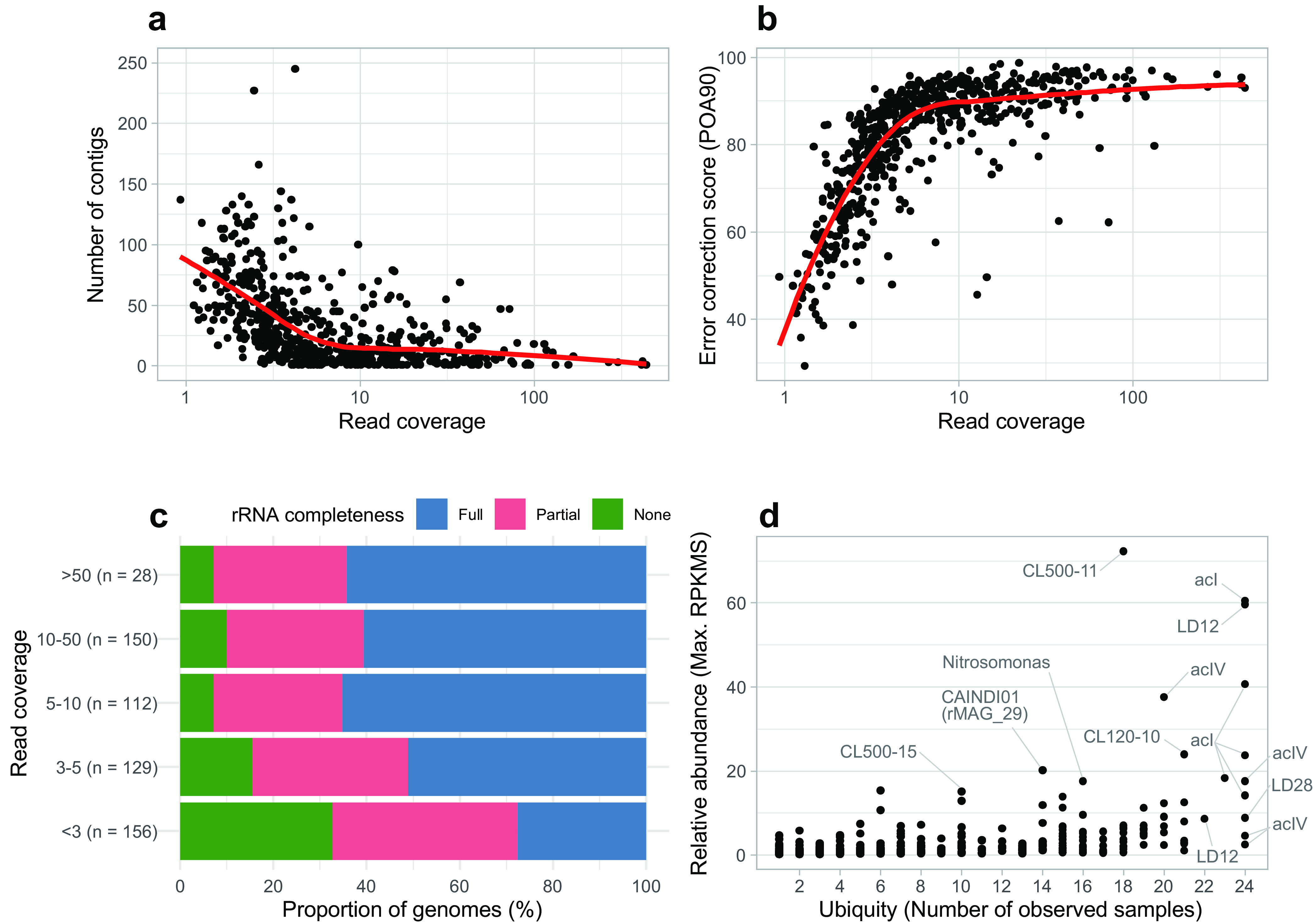
Overview of the 575 rMAGs. Each point represents an individual rMAG. (a and b) Distribution of the number of contigs (a) and error correction scores (POA90; proportion of open reading frames [ORFs] with >90% of the length being aligned to the reference database) (b) plotted against the read coverage. Solid red lines represent local regression (loess). Read coverage was defined as the average short-read coverage in the representative sample for each rMAG. (c) Proportion of rMAGs with different rRNA gene (i.e., 5S, 16S, and 23S) completeness grouped by read coverage value. (d) Ubiquity-abundance plot of the rMAGs. Relative abundance was defined as maximum reads per kilobase of genome per million reads sequenced (RPKMS) recorded among the 24 samples (i.e., those recorded in the representative sample of the rMAG). Ubiquity was defined as the number of samples in which short reads were mapped to >50% of the length of the rMAG sequence. Abundant and ubiquitous members are labeled. Detailed statistics for the rMAGs are available in Table S2.

The 24 samples were associated with broad physicochemical conditions. Thermal stratification occurred from May to December, and the prokaryotic cell abundance was 0.82 to 4.30 (average = 2.00) ×10^6^ cells mL^−1^ (see Table S1 in the supplemental material). For each of the samples, 10.9- to 27.5-Gb long reads (*N*_50_ = 4,360 to 5,807 bp) were assembled, and the resulting contigs were polished using 7.0- to 9.3-Gb short reads (Table S1 and Fig. S1). From the 24 polished contig sets, our pipeline generated 575 nonredundant representative/reference MAGs (rMAGs) covering 21 phyla of bacteria and archaea (Table S2). The number of contigs, the proportion of open reading frames (ORFs) with >90% of the length being aligned to the reference database (POA90) (indel correction score; see Materials and Methods for detail), and completeness of the rRNA genes all showed better results in rMAGs with higher short-read coverage (Fig. 1a to c). For each of the 24 samples, 45.4% to 72.1% (mean = 60.4%) of the short reads were mapped to any of the 575 rMAGs (Fig. S2), indicating that the rMAGs accounted for the majority of the extracted DNA. A ubiquity-abundance plot (Fig. 1d) demonstrated that the rMAGs included common freshwater bacterioplankton lineages known to dominate in Lake Biwa (12, 13, 29). The relative abundance of the rMAGs revealed their diverse distribution pattern across the months and depths (Fig. S3).

10.1128/msystems.00433-22.1 FIG S1 Assembly and binning pipeline in the present study. Only parameters required to reproduce the analysis are shown. Download FIG S1, PDF file, 0.3 MB. Copyright © 2022 Okazaki et al. 2022 Okazaki et al. https://creativecommons.org/licenses/by/4.0/ This content is distributed under the terms of the Creative Commons Attribution 4.0 International license.

10.1128/msystems.00433-22.2 FIG S2 Proportion of short reads mapped to the 575 rMAGs. Download FIG S2, PDF file, 0.1 MB. Copyright © 2022 Okazaki et al. 2022 Okazaki et al. https://creativecommons.org/licenses/by/4.0/ This content is distributed under the terms of the Creative Commons Attribution 4.0 International license.

10.1128/msystems.00433-22.3 FIG S3 Relative abundances (RPKMS) of representative rMAGs across the 24 samples. Asterisks indicate the top 15 rMAGs with the highest nonsynonymous SNV ratios (delineated in Fig. 3a; *n* = 15). Gray cells indicate an RPKMS of 0 (i.e., not detected). The stratification period was from May to December. Epi., Epilimnion; Hypo., Hypolimnion. Download FIG S3, PDF file, 0.1 MB. Copyright © 2022 Okazaki et al. 2022 Okazaki et al. https://creativecommons.org/licenses/by/4.0/ This content is distributed under the terms of the Creative Commons Attribution 4.0 International license.

10.1128/msystems.00433-22.9 TABLE S1 Environmental parameters and sequencing statistics of the 24 samples. Download Table S1, XLSX file, 0.01 MB. Copyright © 2022 Okazaki et al. 2022 Okazaki et al. https://creativecommons.org/licenses/by/4.0/ This content is distributed under the terms of the Creative Commons Attribution 4.0 International license.

10.1128/msystems.00433-22.10 TABLE S2 Statistics and analytical results for each of the 575 rMAGs. Note that those with >10× short-read coverage in the representative sample (*n* = 178) were analyzed mainly for their microdiversity (see column S in the Excel sheet). Download Table S2, XLSX file, 0.6 MB. Copyright © 2022 Okazaki et al. 2022 Okazaki et al. https://creativecommons.org/licenses/by/4.0/ This content is distributed under the terms of the Creative Commons Attribution 4.0 International license.

### SNVs and SVs detected in the rMAGs.

**FIG 2:**
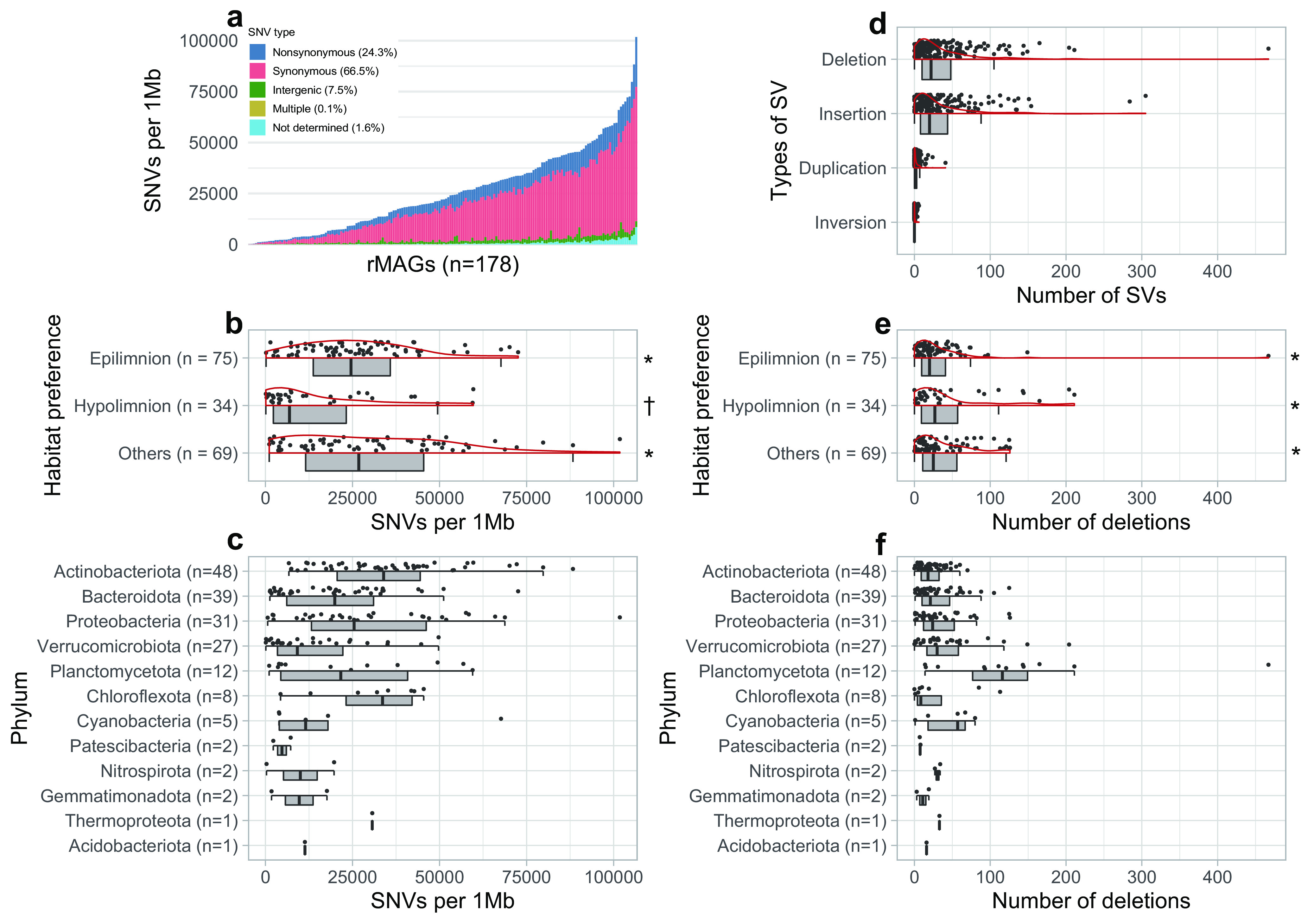
Overview of SNVs and SVs among the 178 rMAGs with >10× short-read coverage. Data were from the representative sample for each rMAG. (a) Each bar represents an individual rMAG, sorted by the number of SNVs per 1 Mb. SNV types determined by inStrain are shown in different colors. The mean proportion of each SNV type among the rMAGs is shown in the color legend. (b to f) Individual rMAGs are represented by each point. (b and c) Distribution (half-violin-boxjitter plot) of the number of SNVs per 1 Mb grouped by habitat preference (b) and phylum (c). (d) Distribution of the number of the four types of SVs in an rMAG. (e and f) Distribution of the number of deletions in an rMAG grouped by habitat preference (e) and phylum (f). The same symbol (* or †) in panel b or e indicates no significant difference (*P* > 0.05 in the Wilcoxon rank sum test) among the groups.

SNVs and SVs were profiled for the 178 rMAGs with >10× short-read coverage in the representative sample. The results revealed the broad spectrum of genomic microdiversity across the rMAGs (Fig. 2). The number of SNVs per 1 Mb ranged from 100 to 101,781 and significantly varied among the habitat preferences (Fig. 2b). Among the four types of SVs detected, insertion (0 to 305 sites per rMAG) and deletion (0 to 467) dominated over duplication (0 to 41) and inversion (0 to 6) (Fig. 2d). The numbers of insertions and deletions were strongly correlated (Pearson’s *r* = 0.925), while they showed weaker correlations (Pearson’s *r* = 0.241 and 0.285) with the number of SNVs (Fig. S4). Unlike SNVs, the number of SVs (deletions) did not significantly vary among the habitat preferences (Fig. 2e). Both the numbers of SNVs and the numbers of SVs (deletions) varied among and within the phyla (Fig. 2c and f).

10.1128/msystems.00433-22.4 FIG S4 Pairwise plots (upper right panels) among the number of insertions, deletions, and SNVs per 1 Mb. Data were from the representative sample for each rMAG. A solid line represents local regression (loess); 95% confidence intervals are shaded gray. Histograms on diagonal panels indicate the distribution of each parameter. Bottom left panels show the Pearson correlation (*r*), with triple asterisks and double asterisks indicating *P* values of <0.001 and <0.01, respectively. Download FIG S4, PDF file, 0.1 MB. Copyright © 2022 Okazaki et al. 2022 Okazaki et al. https://creativecommons.org/licenses/by/4.0/ This content is distributed under the terms of the Creative Commons Attribution 4.0 International license.

### Genes involved in SNVs and SVs.

**FIG 3:**
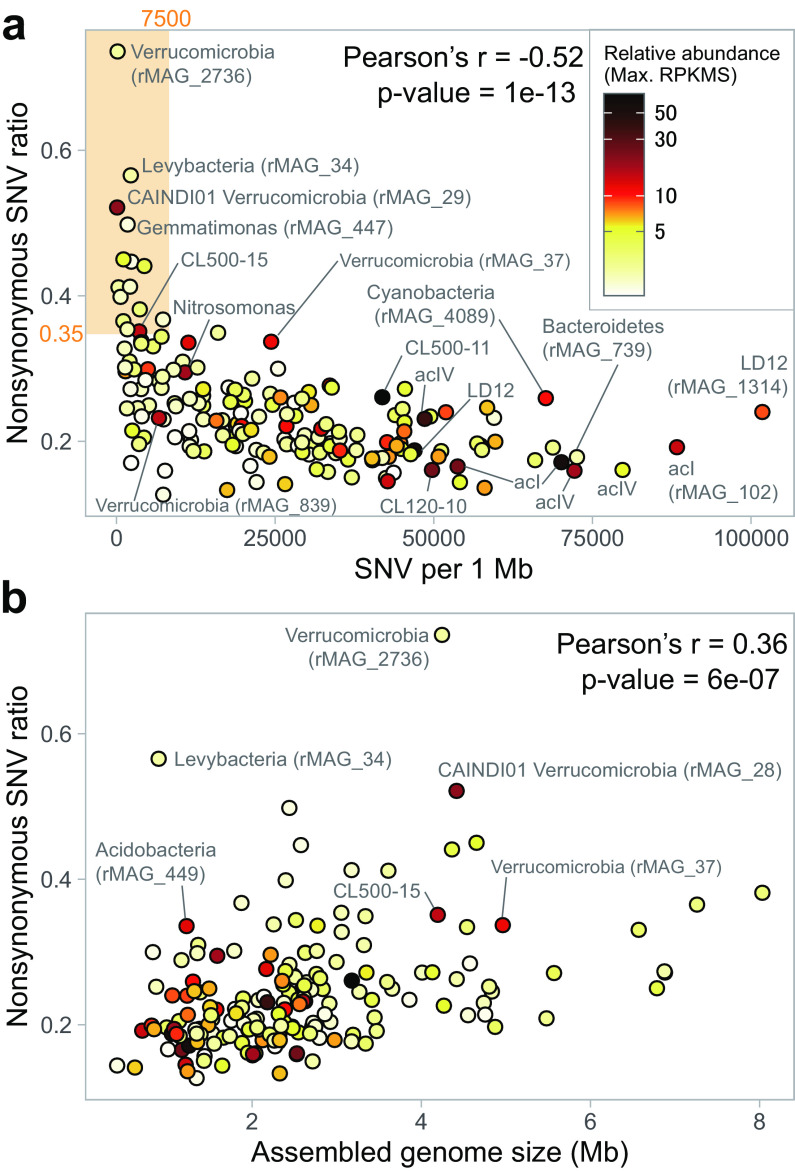
Nonsynonymous SNV ratio of each rMAG plotted against the number of SNVs per 1 Mb (a) and assembled genome size (b). Data were from the representative sample for each rMAG. Plot color indicates the relative abundance (maximum RPKMS) of each rMAG defined as described in the legend for Fig. 1. Representative rMAGs with a high relative abundance or nonsynonymous SNV ratio are labeled. The orange-shaded area in panel a delineates the 15 rMAGs with outstandingly high nonsynonymous SNV ratios (>35%) and a low number of SNVs (<7,500 per 1 Mb).

**FIG 4:**
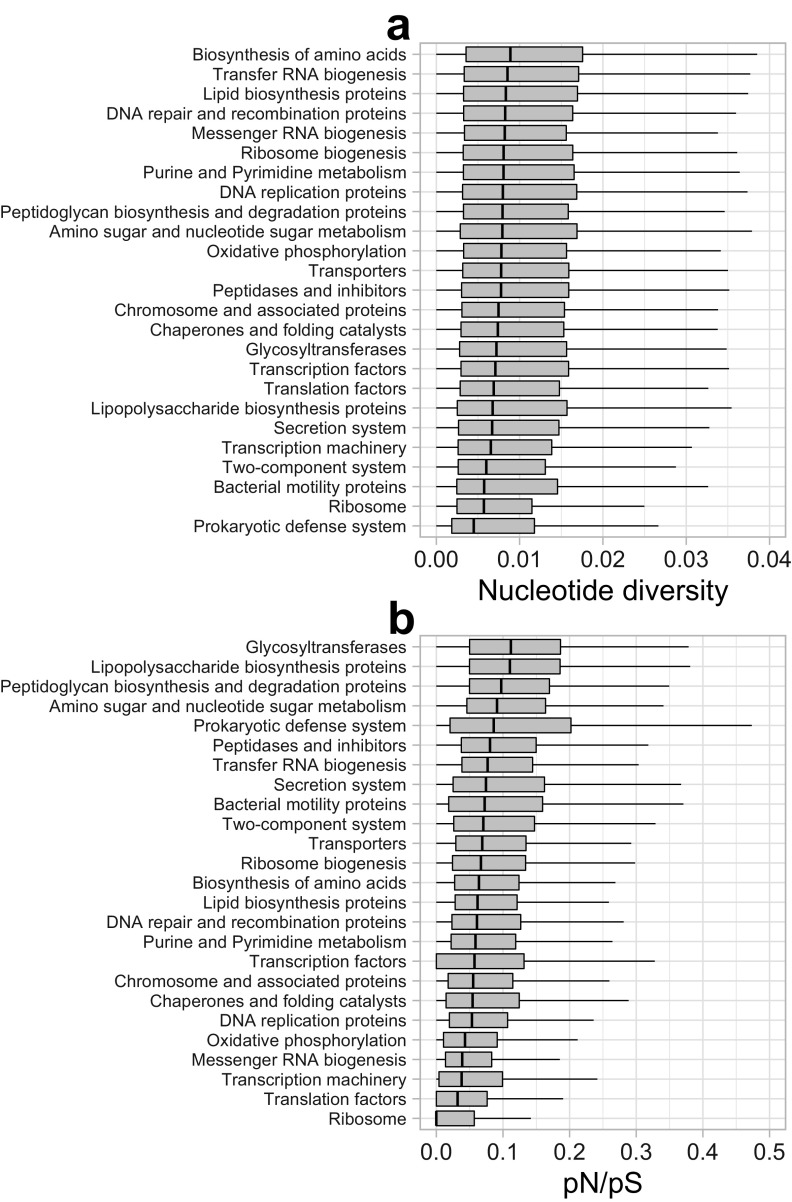
Box plots indicating the distribution of the nucleotide diversity (a) and pN/pS (b) of genes among the 178 high-coverage rMAGs grouped by gene categories. Data were from the representative sample for each rMAG. The categories are sorted by the median. Both nucleotide diversity and pN/pS were determined by inStrain. The nucleotide diversity of a gene is defined as a gene-wide average of base-wise nucleotide diversity expressed as 1 – (*F_A_*^2^ + *F_C_*^2^ + *F_G_*^2^ + *F_T_*^2^), where *F_X_* is the frequency of base *X* in the given nucleotide position.

On average, 66.5%, 24.3%, and 7.5% of SNVs were synonymous, nonsynonymous, and intergenic, respectively (Fig. 2a). The nonsynonymous SNV ratio exhibited a negative correlation with the SNV numbers, and exceptionally high ratios (>35%) were observed among rMAGs (*n* = 15) with low SNV numbers (<7,500 per 1 Mb) (Fig. 3a). The nonsynonymous SNV ratio was positively correlated with genome size (Fig. 3b). Gene-resolved SNV frequency and the ratio of nonsynonymous to synonymous polymorphism rates (pN/pS) exhibited differences among different functional categories (Fig. 4).

**FIG 5:**
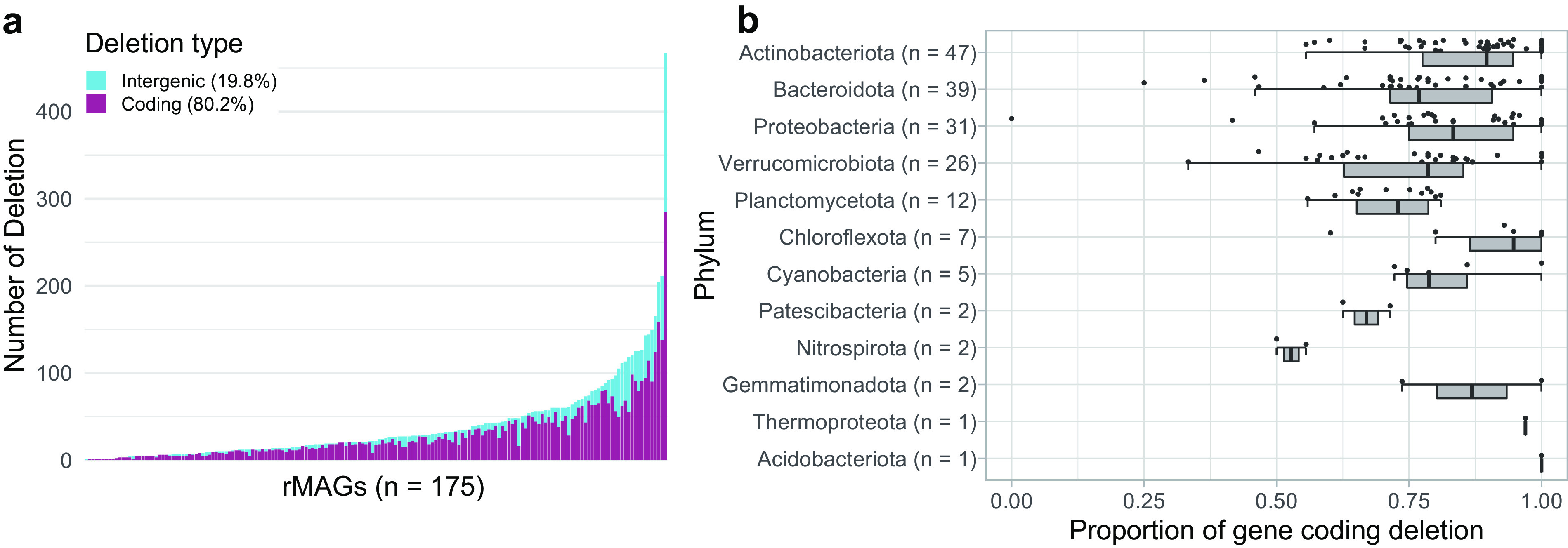
Overview of deletions among rMAGs. Three rMAGs with no deletions were removed from the analysis; the remaining 175 high-coverage rMAGs are shown. Data were from the representative sample for each rMAG. (a) Each bar represents an individual rMAG, sorted by the number of deletions. Coding (i.e., overlapping with a gene coding region) and intergenic deletions are shown in different colors. The mean proportion of each deletion type among the rMAGs is shown in the color legend. (b) Distribution of the proportion of gene coding deletions grouped by phylum. Each point represents an individual rMAG.

**FIG 6:**
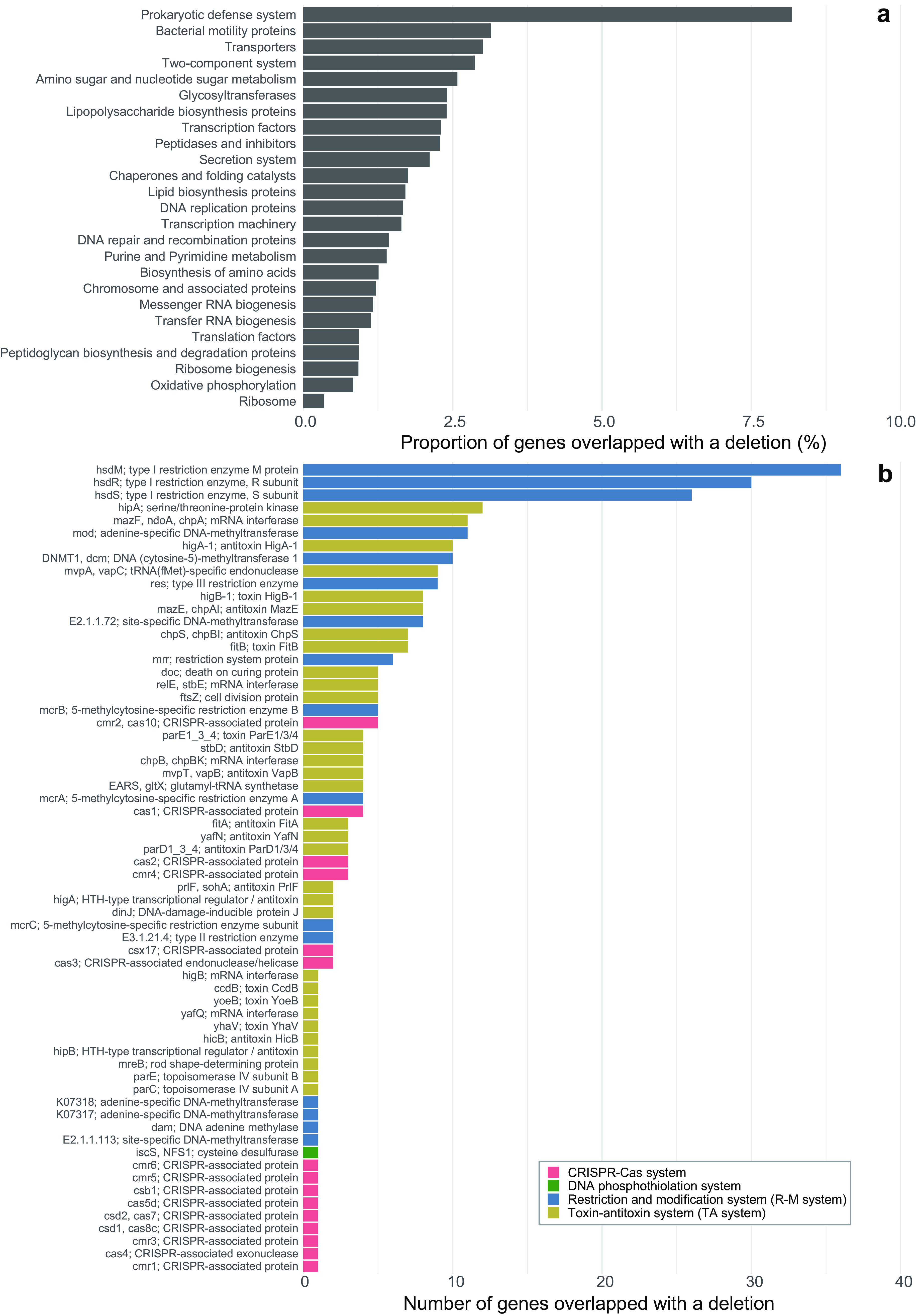
Genes overlapped with a deletion among the 178 high-coverage rMAGs. Data were from the representative sample for each rMAG. (a) Proportion of genes overlapped with a deletion, grouped by gene categories. The same data but shown as the number of genes are available in Fig. S6. (b) Number of prokaryotic defense system genes overlapped with a deletion, with the color indicating the type of defense system.

Among the four types of SVs, we further focused on deletions, because deletion was the most prevalent SV type (Fig. 2d) and genes overlapped with a deletion can be simply characterized on a genome. The second reason is not the case for insertion, in which the involved genes appear in the mapped long reads, which are unpolished and unannotated. Among the 9,471 deletions detected in the 178 rMAGs, 35.2% were <100 bp, followed by a long-tail distribution, with 31.7% and 3.4% being over 1 kb and 10 kb, respectively (Fig. S5). On average, 80.2% of deletions overlapped with a gene coding region (Fig. 5a), and the proportion of gene coding deletions showed a wide range within and among the phyla (Fig. 5b). Gene coding deletions were most frequently overlapped with transporter genes, which reflects the large number of transporter genes in the rMAGs (Fig. S6). Normalized by the gene counts, genes associated with the prokaryotic defense system were most often (>8% of the genes) overlapped with a deletion (Fig. 6a). Among the genes affiliated with the prokaryotic defense system, those associated with the type I restriction and modification (RM) system were most abundant in deletions, followed by genes associated with toxin-antitoxin (TA) systems, other RM systems, and CRISPR-Cas systems (Fig. 6b).

10.1128/msystems.00433-22.5 FIG S5 Length distribution of the 9,471 deletions detected among the 178 rMAGs. Data were from the representative sample for each rMAG. Download FIG S5, PDF file, 0.1 MB. Copyright © 2022 Okazaki et al. 2022 Okazaki et al. https://creativecommons.org/licenses/by/4.0/ This content is distributed under the terms of the Creative Commons Attribution 4.0 International license.

10.1128/msystems.00433-22.6 FIG S6 The number of genes in total (right) and those overlapped with a deletion (left) in each gene functional category among the 178 rMAGs analyzed. Data were from the representative sample for each rMAG. Gene categories are sorted by the number of genes overlapped with a deletion. Download FIG S6, PDF file, 0.1 MB. Copyright © 2022 Okazaki et al. 2022 Okazaki et al. https://creativecommons.org/licenses/by/4.0/ This content is distributed under the terms of the Creative Commons Attribution 4.0 International license.

## DISCUSSION

### Long-read metagenomes generated an ecosystem-wide, high-quality prokaryotic genome collection from Lake Biwa.

Long-read metagenomics successfully reconstructed high-quality MAGs (Fig. 1) representing the majority of the prokaryotic diversity in the lake across seasons and depths (Fig. 1d; see Fig. S2 in the supplemental material), which was not possible by conventional short-read metagenomics in Lake Biwa (13) or other deep freshwater lakes (30–32). The MAGs included 29 closed assemblies, including the first circular representatives of predominant hypolimnetic bacterioplankton lineages, namely, *Chloroflexi* CL500–11 (rMAG_38), *Nitrosoarchaeum* (rMAG_256), *Verrucomicrobia* CL120–10 (rMAG_78), “*Candidatus* Kapabacteria” LiUU-9–330 (rMAG_1819), and a member of *Nitrosomonadaceae* (rMAG_1024) (33, 34).

We should note that we aimed to generate continuous consensus contigs by merging results from different assemblers and samples rather than disjoining microvariants of each genotype. We took this “consensus-first” approach because our subsequent aim was to detect microdiversity masked by the consensus assembly through read mapping. Caveats in analyzing our rMAGs are that they may not represent a single genotype existing in the environment and they may still contain base errors left unpolished due to inadequate short-read coverage. The POA90 score suggested that fragmented ORFs introduced by uncorrected indel error are common in the majority of genomes with <10× short-read coverage (Fig. 1b). In light of these limitations, we designate our MAGs as rMAGs (representative/reference MAGs) to differentiate them from those generated by conventional short-read metagenomics and focused on those with >10× short-read coverage (*n* = 178) for further investigation. In the downstream analyses (Fig. 2 to 6), we considered only SNVs and SVs in the representative sample for each rMAG rather than concatenating the results from multiple samples. We took this approach because the concatenation would introduce biases in comparing genomes and genes due to the uneven number of high-coverage (>10×) samples among the rMAGs.

The general trend that a higher read coverage resulted in a higher-quality rMAG (Fig. 1) suggests that our sequencing effort (Table S1) was unsaturated and that deeper sequencing would generate a greater number of high-quality rMAGs. However, read coverage alone was not sufficient to reconstruct a high-quality rMAG. For example, an rMAG of LD12 (“*Candidatus* Fonsibacter”), which is among the most abundant freshwater bacterioplankton lineages (35, 36), was fragmented and lacked rRNA genes, despite their extremely high read coverage (>400× in short reads). Members of *Pelagibacterales* (also known as the SAR11 clade), including LD12, harbor high genomic microdiversity in the flanking region of the rRNA gene operon that is presumably responsible for immunity against their phage (21, 35, 37, 38). Our results indicate that long-read sequencing generally deals well with “the great metagenomics anomaly” (5) but is still unable to solve the issue in extreme cases. Nonetheless, rMAGs provided an unprecedentedly high-quality lake prokaryotic genome collection, which allowed ecosystem-wide exploration of their genomic microdiversity through read mapping.

### Broad spectrum of genomic microdiversity resolved by SNVs and SVs.

We found more than 1,000-fold differences in the SNV frequency across the rMAGs (Fig. 2a), which is in line with a report on another freshwater system (39). The dominance of synonymous SNVs (Fig. 2a) is also in agreement with previous works in freshwater (39) and marine (21, 40) systems, supporting the idea that the bacterioplankton assemblage is generally under purifying selection, with most of the nucleotide variation being neutral. The positive correlation between the nonsynonymous SNV ratio and genome size (Fig. 3b) agrees with the hypothesis that genome streamlining is associated with strong purifying selection (41–43). We further found that the frequency of SNVs was lower (Fig. 2b) and also more temporally stable (Fig. S7) in genomes of hypolimnion inhabitants than that in genomes of epilimnion inhabitants. These results imply a lower mutation rate in the deeper water layer, presumably due to the lower UV-induced oxidative stress or the lower biological productivity owing to the lower temperature and resource availability in the hypolimnion.

10.1128/msystems.00433-22.7 FIG S7 Distribution (half-violin-boxjitter plot) of the range (maximum value to minimum value) of the nucleotide diversity of each rMAG (represented by each point) during the stratification period (May to December). The rMAGs for which the nucleotide diversity could be calculated for more than 4 of the 8 months in each of the water layers were included in the analysis. The range was significantly broader in the epilimnion than in the hypolimnion, according to the Wilcoxon rank sum test. Download FIG S7, PDF file, 0.1 MB. Copyright © 2022 Okazaki et al. 2022 Okazaki et al. https://creativecommons.org/licenses/by/4.0/ This content is distributed under the terms of the Creative Commons Attribution 4.0 International license.

One of the major achievements of the present study was the detection of SVs in a metagenomic sample facilitated by long-read mapping. Compared to the SV analysis for an isolated clonal genome, that for metagenomic assembly generates more complex outputs as it refers to a consensus assembly derived from a highly heterogeneous population. Notably, our approach was not efficient in detecting SVs with a high allele variation or frequency, because such a highly heterogeneous region often eludes metagenomic assembly. Conversely, our approach cannot detect mobile elements that did not show heterogeneity within the 24 samples. Given these technical limitations, our goal was not to resolve all SVs but rather to discover patterns of SV distribution among environmental prokaryotic genomes under the same methodological criteria. Indeed, most SVs in a genome were consecutively detected across samples of different months (Fig. S8a), supporting the reproducibility and robustness of our analysis.

10.1128/msystems.00433-22.8 FIG S8 SVs visualized on Integrative Genomics Viewer (IGV). The locations of SVs on an rMAG sequence are indicated by blue and red symbols based on the variant call format (.vcf) file generated by Sniffles for each sample. The allele frequency (AF) of each SV is represented by the height of the red-colored part against the blue-colored part. Shaded SVs are those filtered out by Sniffles software due to poor or inconsistent support with read mapping results. (a) Temporal profile of SVs in four representative rMAGs that showed continuously high read coverage during the study period in either of the water layers. The top two rMAGs are single-contig bins, while the longest contig is displayed for the other two rMAGs. SV positions in each sequence were chronologically sorted from top (May 2018) to bottom (April 2019). Most SVs were continuously present during the study period. (b) Deletions on intergenic tandem repeats in rMAG_354 (*Phycisphaerales* of the phylum *Planctomycetota*). The top panel indicates the SV profile in a representative genomic region of the rMAG. The blue boxes shown below the SVs denote ORFs. Black arrows indicate intergenic deletions; red circled numbers indicate those involving tandem repeats, for which enlarged visualizations are shown in the bottom panels. Nucleotide sequences and repeat motifs (blue lines below the sequence) are shown in the enlarged panels. (c) Deletions involving a CRISPR-Cas system. In the top case (rMAG_305), the deletion was continuously detected from October to February in the epilimnion, with decreasing allele frequency. The deletion also included TA system proteins downstream of the CRISPR. In the bottom case (rMAG_1349), the allele frequency shifted more quickly from 0.15 in August to 0.83 in October. The coverage track and pileup of the mapped long reads are shown for each sample and indicate that many reads were aligned to the SV region in August while most of the reads bridged the edges of the SV region in October. (d) SVs associated with variation in CRISPR spacer sequences. Both rMAGs showed a shift of SV pattern in the CRISPR sequences during consecutive months in the hypolimnion. SV type, allele frequency, and the sequence involved in each SV are shown in an orange box, in which CRISPR repeat sequences are shaded yellow. Note that sequences shown for insertion were predicted using sequences of the mapped raw long reads and thus were unpolished (i.e., error prone). Download FIG S8, PDF file, 1.2 MB. Copyright © 2022 Okazaki et al. 2022 Okazaki et al. https://creativecommons.org/licenses/by/4.0/ This content is distributed under the terms of the Creative Commons Attribution 4.0 International license.

As with SNVs, we observed significant variation in SV frequency among the rMAGs (Fig. 2d). The relationship between the number of SNVs and SVs was weak because several rMAGs had an extremely high number of SVs (Fig. S4). Notably, members of *Planctomycetota* harbored disproportionally high numbers of SVs (Fig. 2f) and a lower frequency (55.9% to 81.0%) of coding deletions (i.e., those overlapping with an ORF) than the average (80.2%) (Fig. 5b). Further investigation found that their noncoding deletions were often associated with intergenic tandem repeats (Fig. S8b). Such duplications and deletions can be introduced by slippage of DNA polymerase during replication and can regulate the transcriptional activity or act as a recombination site (44). Planctomycetes generally harbor a large genome with a high number of genes with unknown functions (45). A recent exploration of freshwater planctomycete MAGs reported a correlation between their genome size and intergenic nucleotide length (46). Together, their intergenic plasticity might play an essential role in maintaining their genomic integrity. Although characterization of individual SVs is beyond the scope of the present study, overall, our long-read-resolved ecosystem-wide analysis reveals the ubiquity of SVs in environmental prokaryotic genomes and sheds light on their role in regulating genomic structure and function.

### Genetic bottleneck as a major constraint of genomic microdiversity.

The negative relationship between SNV frequency and their nonsynonymous rate (Fig. 3a) suggests that stronger purifying selection acts on a genome in which more mutations are accumulated. Based on this assumption, the lineages with a high nonsynonymous SNV ratio and a low number of SNVs may have experienced a recent population bottleneck and not mutated sufficiently to be negatively selected. In other words, their diversification process might still be dominated by random drift or positive selection. Indeed, the top 15 rMAGs with the highest nonsynonymous SNV ratio (delineated in Fig. 3a) were either continuously rare in the hypolimnion or mostly rare but predominant in a short period (boom-and-bust) in either of the water layers (Fig. S3). The former case could be the consequence of the low growth and mutation rates in the hypolimnion, which makes the genome diversification of these lineages slow enough to be observed before purifying selection dominates. Notably, among these cases, the highest nonsynonymous SNV ratio was observed in rMAG_34, which is affiliated with “*Candidatus* Levybacteria” (OP11), a member of the candidate phyla radiation (CPR) (47). Recently, a comprehensive exploration of freshwater CPR MAGs (48) reported exceptionally high average nucleotide identity (ANI) (99.53%) between levybacterial MAGs reconstructed from Lake Biwa (13) and Lake Baikal (31) metagenomes. We confirmed that our levybacterial rMAG also belonged to the same species (ANI > 99.5% to both). Collectively, it is possible that “*Candidatus* Levybacteria” recently migrated from the Eurasian continent to Lake Biwa and that their genomic microdiversity was still constrained by the genetic bottleneck.

Among the latter (boom-and-bust) cases, prominent examples were two verrucomicrobial rMAGs (rMAG_2736 and rMAG_29), which had extremely low numbers of SNVs and SVs (Fig. 3a and Table S2) and transiently dominated in either of the water layers (Fig. S3). Both rMAGs were circular, indicating that long-read metagenomes generate a complete assembly unless hampered by high microdiversity or low read coverage. The boom-and-bust dynamics of *Verrucomicrobia* agrees with the general assumption that they are opportunistic strategists rapidly responding to a supply of carbohydrates (49, 50). Notably, rMAG_29 (taxonomically assigned to the genus “CAINDI01” by the GTDB) was among the most abundant bacterioplankton lineages in the lake during their bloom (Fig. 1d and Fig. S3), with their relative abundance (reads per kilobase of genome per million reads sequenced [RPKMS]) increasing more than 12-fold in just 1 month (1.39 in November to 16.92 in December). Because their bloom was observed from May to June and from December to January in the hypolimnion (Fig. S3), their growth was likely triggered by a supply of polysaccharides exuded from sinking phytoplankton cells derived from the spring and autumn algal blooms in the epilimnion, as observed in a previous study of the lake (51). Taken together, the ecological strategy of CAINDI01 (to rapidly exploit intermittent resources) produced periodic genetic bottlenecks and effectively eluded selective processes, which resulted in their extremely low genomic microdiversity in the lake despite their quantitative dominance. Interestingly, CAINDI01 contained as many as 236 transposase genes (annotated by Prokka), but none of them were associated with SVs, except for an inversion involving IS*21* transposases (data not shown). The results further suggest that their rapid population turnover prevented invasions of mobile genetic elements (MGEs). Collectively, we conclude that a genetic bottleneck is a primary factor constraining genomic microdiversification.

Conversely, the extent of genomic microdiversification may be used to predict the existence or absence of a recent bottleneck event. For instance, rMAG_739 (*Chitinophagaceae* of the phylum *Bacteroidetes*) was the fourth-most SNV-rich rMAG, with a low nonsynonymous rate (Fig. 3a), despite the fact that these bacteria were detectable only from June to October in the epilimnion (Fig. S3). These results suggest that they did not experience a recent genetic bottleneck and thus are allochthonous, presumably maintaining their large genetic pool in the inflowing river, sediment, or the water column horizontally distant from our sampling site. It should also be noted that no sign of a recent bottleneck event was found among common and abundant freshwater bacterioplankton lineages (e.g., LD12, acI, acIV, and CL500–11). Interestingly, the two most SNV-rich members, rMAG_1314 and rMAG_102, were hypolimnion-dominating species of LD12 and acI, respectively, rather than those dominant in the epilimnion (i.e., rMAG_300 and rMAG_28) (Fig. 3a and Fig. S3). The results further support the idea that persistent rather than dominant populations exhibit higher intrapopulation sequence variation (52). Given that the hypolimnion accounts for a larger part of the lake water volume and is a less competitive habitat than the epilimnion, we hypothesize that hypolimnion inhabitants are more likely to sustain a larger and more stable population and thus are less constrained by a population bottleneck than epilimnion inhabitants.

### Phage predation as a major driving force of genomic microdiversification.

The lowest pN/pS ratio in housekeeping genes involved in replication, transcription, translation, and oxidative phosphorylation (Fig. 4b) agreed with that of a previous study in the Baltic Sea (25) and indicated that the genes involved in core functions are under stronger purifying selection. In contrast, high pN/pS ratios were observed among genes potentially involved in cell surface structural modification, namely, glycosyltransferases, lipopolysaccharide biosynthesis, and peptidoglycan biosynthesis proteins (Fig. 4b). Hypervariability of such genes has been observed in genomes of ubiquitous marine and freshwater bacterioplankton and is considered beneficial in evading the host recognition system of their phage (7–9). Our results further demonstrate that these traits are universal in the ecosystem and suggest that phage predation is the most prevalent selective pressure generating amino acid-level gene diversity.

The SV profiling demonstrated that deletion was overrepresented in genes involved in prokaryotic defense systems, namely, RM systems, TA systems, and CRISPR-Cas systems (Fig. 6a). Among them, the three proteins making up the type I RM system (R, M, and S) were the most represented (Fig. 6b). A previous metaepigenomic exploration revealed the diversity of DNA methylated motifs and methyltransferase genes among Lake Biwa bacterioplankton assemblages (53). Interestingly, the study reported that a corresponding pair of a methylated motif and a methyltransferase gene is often absent in MAGs, which could be attributable to the incompleteness of MAGs or to the limited sensitivity of the method. Further, the study found that the ratio of methylation in each motif in a genome varied considerably, from 19% to 99%, for which the authors reasoned reflected the methodological limitation of modification detection power (53). Our results introduce another possible explanation for these observations: the mobility of RM-related genes within a sequence-discrete population might have resulted in the heterogeneous recovery of methylated motifs or methyltransferase genes in a MAG. The variable nature of epigenetic modification proposes another layer of genomic microdiversity, which will be key to revealing the mechanism behind the virus-host arms race.

The next most represented defense genes in deletions were those involved in TA systems (Fig. 6b), which can also act as an antiphage system (54). Recent experimental work has demonstrated that the mobility and rapid turnover of genes involved in intracellular defense machinery are essential mechanisms to maintaining the core genome in the face of phage predation (55). Our results that RM and TA systems are highly mobile (Fig. 6b) suggest the prevalence of such mechanisms in the ecosystem. In addition, SNV analysis revealed that the prokaryotic defense system was the gene category with the lowest nucleotide diversity (Fig. 4a) and among the highest pN/pS ratios (Fig. 4b), which implies that the defense genes are positively selected by phage predation. Meanwhile, both RM and TA systems can behave as selfish and addictive elements and are prone to be horizontally transferred with an MGE (54, 56, 57). Their beneficial and parasitic aspects are not mutually exclusive, and the relative contribution of the two remains unresolved. Thus, we cannot rule out the possibility that some defense genes are rather parasitic and nonbeneficial or even detrimental for the host. In any case, these genes are among the most prevalent mobile genes generating genomic heterogeneity within a sequence-discrete population.

Although not as frequent as RM and TA systems, we also found deletions associated with genes involved in the CRISPR-Cas system (Fig. 6b). Further investigation revealed individual cases in which the whole CRISPR-Cas system was involved in a deletion, and one of them further included TA system genes (Fig. S8c). Experimental studies have suggested that the CRISPR-Cas system can disseminate horizontally (58, 59) and is sometimes encoded in an MGE, which facilitates not only adaptive immunity against phages but also inter-MGE competition and guided transposition of the MGE (60–62). Our results provide evidence of the mobility of the CRISPR-Cas system in an ecosystem, although it remains unknown whether it is beneficial or parasitic for the host.

Finally, we note that our monthly investigation revealed a shift in the allele frequency of deletions or insertions involving the CRISPR-Cas system and CRISPR spacers during the study period (Fig. S8c and d). The results suggest monthly turnover of the population composition driven by the virus-host arms race. Such a rapid shift in population composition has been demonstrated from the virus side in the marine system (22). Our results are the demonstration from the host side and propose the significance of not only sympatric but also temporal microdiversity. In summary, our ecosystem-wide investigation of SNVs and SVs suggests that phage predation is the major driving force of genomic microdiversification among the environmental microbial assemblage. The key question for future works is whether and how the mobility of defense genes is beneficial for the host, for which the microdiversity of the counteracting viral genome must be explored.

### Conclusions.

Our ecosystem-wide high-resolution approach combining spatiotemporal sampling and long- and short-read metagenomics resulted in two major achievements. First, we presented a collection of high-quality MAGs covering the majority of the prokaryotic diversity in a deep freshwater lake, which will be a valuable reference for future studies in freshwater microbial ecology. Then, the broad spectrum of SNVs and SVs masked in the MAGs were detected by short- and long-read mapping, respectively, which is the second and greater achievement of this work. Based on the results, we conclude that genomic microdiversification is driven primarily by viral load and constrained by genetic bottlenecks.

We also demonstrated the performance and limitation of our “consensus-first” approach (Fig. 1). To push the consensus-first approach further, future works can consider gaining a deeper sequencing depth (for instance, using the PromethION platform [63, 64]) and obtaining longer sequencing reads with a more sophisticated DNA extraction method (65). Alternative possible approaches include genome-free metagenomics, which directly handles pan-metagenomic graphs without the prerequisite of a linear genomic assembly (66). The ultimate approach will be a strain-resolved assembly, which usually requires an isolated culture or single cell but was recently accomplished in a metagenomic assembly using highly accurate long reads (i.e., PacBio HiFi reads) and high-throughput chromosome conformation capture (Hi-C) (20), although it is still too costly for common application.

Lakes are physically separated unique ecosystems and thus harbor genetically isolated microbiomes (67), while those in the marine system are likely distributed globally (40, 68) presumably following the rapid circulation of global surface seawater (69). This implies that we can further perform a comparative study among different lakes, in which each lake can be considered as a replicate or control of an ecosystem. The two main factors affecting genome microdiversification (genetic bottlenecks and virus-host interactions) are both lake specific. The microbiomes in different lakes have different histories of biological interactions under different physicochemical conditions, which would result in different trajectories of genome microdiversification. For instance, we hypothesize that a larger and older lake is less affected by genetic bottlenecks in terms of time and space. That is, the extent of bacterioplankton microdiversification in Lake Biwa (the largest and oldest lake in Japan) might be the greatest among the lakes in the country but might be lower than that of Lake Baikal, the largest and oldest freshwater lake on the earth. Such interlake comparative analyses will be an effective approach to further validate the findings in the present study and to unveil the universal mechanisms in the diversification and evolution of the microbial genome.

## MATERIALS AND METHODS

### Sample collection.

Water samples were collected monthly from May 2018 to April 2019 at a pelagic station (water depth, ca. 73 m) on Lake Biwa (35°13′09.5″N, 135°59′44.7″E) from two water depths, representing the epilimnion (5 m) and the hypolimnion (65 m) (24 samples in total). Vertical profiles of chlorophyll *a* concentration, temperature, and dissolved oxygen were collected using a RINKO CTD profiler (ASTD102; JFE Advantech). The collected lake water was immediately sequentially filtered through a 200-μm mesh, 5-μm polycarbonate filter (TMTP14250; Merck Millipore) and a 0.22-μm-pore-size Sterivex cartridge (SVGP01050; Merck Millipore), using a peristaltic pump system onboard. Filtration was performed until the Sterivex cartridge was clogged (1 to 2.5 L of lake water was filtered for each cartridge), and at least four Sterivex cartridges were collected for each sample. The filters were flash-frozen in a dry ice-ethanol bath, transported to the laboratory on dry ice, and stored at −80°C until further processing. Water samples were collected between 8:00 a.m. and 11:00 a.m. on each sampling day and processed to the freezing step within 1 h after collection. Prokaryotic cell abundance was determined for each sample using a flow cytometer (CytoFLEX; Beckman Coulter) following fixation of the water sample with 1% glutaraldehyde and staining with 0.25× SYBR green solution (S7563; Invitrogen).

### DNA extraction.

DNA was extracted from the Sterivex filters (i.e., 0.22- to 5-μm size fraction) using an AllPrep DNA/RNA minikit (catalog no. 80204; Qiagen) with a modified protocol: the filter paper removed from a Sterivex cartridge was put into a lysing matrix E tube (catalog no. 6914050; MP Biomedicals) with a mixture of 400 μL RLT plus buffer (containing 1% β-mercaptoethanol in accordance with the kit’s protocol) and 400 μL phenol-chloroform/isoamyl alcohol (25:24:1, vol/vol/vol); bead-beating was performed at 3,500 rpm for 30 s (MS-100; TOMY Digital Biology), followed by cooling on ice for 1 min, and then again at 3,500 rpm for 30 s; the supernatant after centrifugation (16,000 × *g* for 5 min at room temperature) was mixed with 500 μL chloroform-isoamyl alcohol (24:1, vol/vol) to remove the residual phenol and then centrifuged again; the supernatant was then used as the loading material for the AllPrep DNA spin column and processed in accordance with the manufacturer’s instructions. The quantity and quality of the DNA were measured using a Qubit double-stranded DNA (dsDNA) HS assay kit (catalog no. Q32851; Thermo Fisher Scientific) and a spectrophotometer (NanoDrop 2000; Thermo Fisher Scientific). Consequently, at least 2 μg purified DNA was obtained from each sample.

### Sequencing.

The extracted DNA was used for both short- and long-read shotgun metagenomic sequencing. For short-read sequencing, the DNA was sheared to 500 bp, on average, using an ultrasonicator (Covaris), and a 24-sample multiplexed library was prepared using an MGIEasy universal DNA library prep set (catalog no. 1000006986; MGI), a circularization kit (catalog no. 1000005259; MGI), and a MGISEQ-2000RS high-throughput sequencing set (catalog no. 1000013857; MGI) with seven cycles of PCR amplification. A 1 × 400 bp single-end sequencing was run using one lane of the MGI DNBSEQ-G400 platform. For long-read sequencing, long DNA molecules were purified using diluted (0.45×) AMPure XP beads, and a sequencing library was prepared using a ligation sequencing kit (LSK-109; Oxford Nanopore). Each of the 24 samples was sequenced by an R9.4.1 flow cell (FLO-MIN106D; Oxford Nanopore) using the Oxford Nanopore GridION platform for 72 h. Base calling was performed using Guppy (v3.2.10; high-accuracy mode).

### Read assembly and contig polishing.

Each of the 24 raw long-read libraries was assembled using two different assemblers: Flye (v2.8; –plasmids –meta) (70) and Raven (v1.5.0) (71). The assembled contigs were polished with long reads using Racon (v1.4.13) (72) and Medaka (v1.0.3) (https://github.com/nanoporetech/medaka) and then with short reads using Pilon (v1.23) (73) and two rounds of Racon. Read mapping for polishing was performed using Minimap2 (v2.17) (74) and Bowtie2 (v2.3.5.1) (75). Quality control of short reads was performed using Cutadapt (v2.5) (76) and fastp (v0.20.0) (77). The detailed workflow and parameters are available in Fig. S1 in the supplemental material.

### Binning and bin curation.

Contigs longer than 2.5 kb were selected using SeqKit (v0.13.2) (78), and their read coverage across the 24 samples was calculated by mapping the quality-controlled short reads using CoverM (v0.4.0; -m metabat) (https://github.com/wwood/CoverM). The coverage table was input to MetaBAT (v2.12.1) (79) and MaxBin (v2.2.7) (80) to bin the contigs from each of the 24 Flye and Raven assemblies. The resulting 18,621 bins, containing redundancy derived from 24 samples (2 depths × 12 months), two assemblers (Flye and Raven), and two binners (MetaBAT and MaxBin) (Fig. S1), were curated by the following procedures. Bins sharing an average nucleotide identity (ANI) of >95% were clustered using FastANI (v1.31) (81) and the hclust function (method = “average”) of R v4.0.0 (https://www.r-project.org/). This resulted in 3,053 bin clusters and 1,595 singletons, hereinafter referred to as superbins. Next, bins in the same superbin were merged as follows. First, the bin quality score (BQS) was determined as (completeness − [5 × contamination]), referring to the output of checkM (v1.1.3) (82) for each bin. Then, bins derived from the same sample (i.e., only different in the assembler or binner) were merged using quickmerge (v0.3), which bridges gaps in one assembly (acceptor) using sequences of another assembly (donor) based on alignment overlaps (83). Starting from the bin with the highest BQS as an acceptor, bins were iteratively merged by providing a donor bin in the order of BQS. For bins with the same BQS, the bin with fewer contigs was selected in priority. The “–hco” parameter was set to 20, which means that the aligned length should be more than 20 times longer than the unaligned length to merge two contigs. Next, if multiple merged bins in the same superbin (i.e., those from different samples) showed a BQS of >50, they were further merged in the same manner as described above. Notably, intersample merges did not always generate a better bin than intrasample merged bins, presumably because of the genomic compositional heterogeneity between samples. Finally, a representative bin was determined for each of the 4,648 superbins by selecting the one with the highest BQS among the original and merged bins.

Among the 4,648 representative bins, 331 consisted of a single contig. Because quickmerge does not consider genome circularity, we attempted their circularization in the following procedure. First, using nucmer (v3.1) (84), the first and last 50 kb of the contig were aligned against the set of contigs in the same superbin to find a “bridging contig” that may close the gap between the ends. Next, if a bridging contig was found, it was supplied as “new_assembly.fasta” to the circlator (v1.5.5) merge function with the “–ref_end 50000” parameter (85). If the circularization was successful, the contig was rotated to start from a *dnaA* gene using the circlator fixstart (–min_id 30) function.

Finally, the 4,648 representative bins were quality filtered at a BQS of >50, followed by dereplication using dRep (v3.0.1; -comp 0 -con 100 -sa 0.95 –SkipMash –S_algorithm fastANI) (86). This final dereplication removed redundancy that eluded the initial superbin clustering, which was not exhaustive due to the limitation of hierarchical clustering of incomplete genomes. The resulting 575 bins were designated representative/reference metagenome-assembled genomes (rMAGs).

### Analysis of rMAGs.

The 575 rMAGs were taxonomically classified using GTDB-Tk (v1.5.0) with the reference data version r202 (87), and the genes were annotated using prokka (v1.14.6) (88) and eggNOG-mapper (v2.1.5) (89). Annotated genes were functionally categorized according to KEGG PATHWAY and KEGG BRITE hierarchies (90) assigned to each gene by eggNOG-mapper. For further analysis, we selected the top 25 functional categories that covered 33% of the genes. To evaluate the frequency of indel errors that eluded polishing, we followed the idea of the IDEEL software, i.e., interrupted open reading frames (ORFs), which are often introduced by a frameshift, were used as an indicator of indel errors (18). Specifically, amino acid sequences for each rMAG predicted by Prodigal (v2.6.3) (91) were aligned against the UniRef90 database (release 2020_06) (92) using DIAMOND blastp (v2.0.6; -k 1 -e 1e−5) (93). Based on the results, the proportion of amino acid sequences in which >90% of the length was aligned to a UniRef90 sequence was determined for each rMAG and designated as the score for the proportion of ORFs with >90% alignment (POA90). Coverage-based abundance relative to the total sequenced DNA in each of the 24 samples was determined as reads per kilobase of genome per million reads sequenced (RPKMS), which was generated by mapping the quality-controlled short reads to the 575 rMAGs using bowtie2 (v2.4.2) (75), followed by counting of mapped and unmapped reads using CoverM (–min-read-percent-identity 92). The habitat preference (epilimnion or hypolimnion) of each rMAG was determined using the metric *P*_epi_, which was defined as the quotient of RPKMS in the epilimnion versus the sum of the values in the epilimnion and hypolimnion [i.e., epilimnion/(epilimnion + hypolimnion)] during the stratification period (May to December). When *P*_epi_ was >0.95 or <0.05, the rMAG was determined as an epilimnion or hypolimnion specialist, respectively (13).

### Analysis of SNVs and SVs.

The gene loci and mapping results (i.e., bam files) generated above were input to inStrain (v1.0.0; profile –database_mode –pairing_filter all_reads), which provides genome- and gene-wide SNV profiles based on the short-read alignment (24). SVs were detected by mapping the raw long reads to the rMAGs using NGMLR (v0.2.7) (26) and inputting the resulting bam files to Sniffles (v1.0.12) (26). Among the five types of SVs reported by Sniffles, deletion, insertion, duplication, and inversion were further analyzed, while translocation was removed in the downstream analyses because translocation can involve multiple contigs in different bins and is hard to interpret in metagenomic data. Subsequently, SVs with low (<0.1) allele frequency (reported by Sniffles) were filtered out. SVs longer than 100 kb were also removed, as they were seemingly artifacts introduced by genome circularity, which Sniffles does not account for.

The representative sample providing the highest short-read coverage among the 24 samples was determined for each rMAG. To remove low-quality data derived from low read coverage, rMAGs that showed >10× short-read coverage in the representative sample (*n* = 178) were selected, and SNVs and SVs in the representative sample were analyzed in detail.

### Data availability.

The raw sequencing reads generated in the present study are available under accession numbers DRR333363 to DRR333410 (BioProject ID PRJDB12736) as summarized in Table S1. Nucleotide fasta files of the rMAGs are available at https://doi.org/10.6084/m9.figshare.19165673.v1.
