# Supplementary figures and images for: Long-Read-Resolved, Ecosystem-Wide Exploration of Nucleotide and Structural Microdiversity of Lake Bacterioplankton Genomes
Source: mSystems. 2022 Aug 8;7(4):e00433-22. doi: 10.1128/msystems.00433-22 (PMC9426551; doi:10.1128/msystems.00433-22)

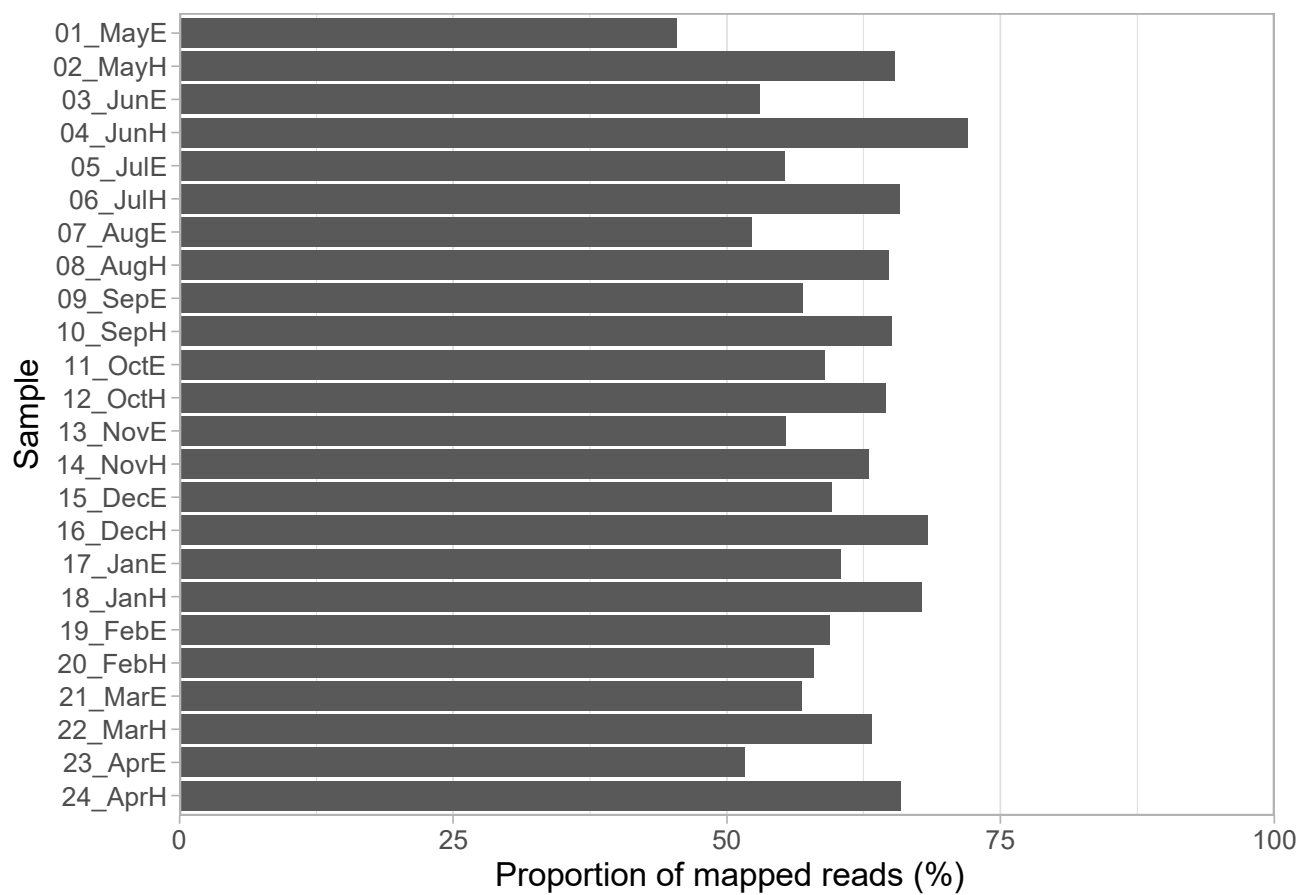

**Figure S2.** Proportion of short reads mapped to the 575 rMAGs.

Supplement: FIG S2 [file msystems.00433-22-s0002.pdf]
