# Supplementary material for: Long-Read-Resolved, Ecosystem-Wide Exploration of Nucleotide and Structural Microdiversity of Lake Bacterioplankton Genomes
Source: mSystems. 2022 Aug 8;7(4):e00433-22. doi: 10.1128/msystems.00433-22 (PMC9426551; doi:10.1128/msystems.00433-22)
Supplement: FIG S3 [file msystems.00433-22-s0003.pdf]

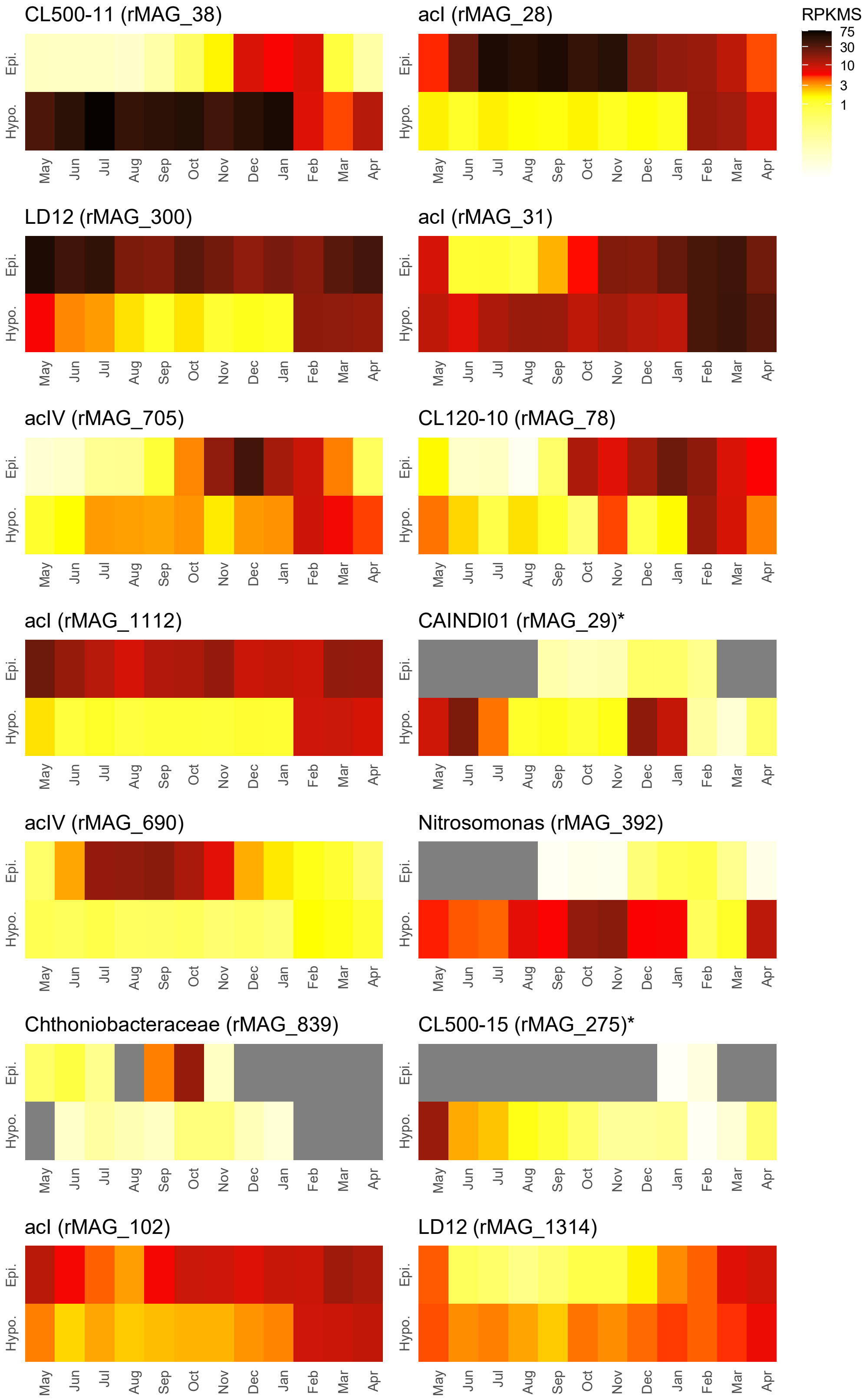
CL500-11 (rMAG_38)
acI (rMAG_28)
RPKMS
75
30
10
3
1
LD12 (rMAG_300)
acI (rMAG_31)
acIV (rMAG_705)
CL120-10 (rMAG_78)
acI (rMAG_1112)
CAINDI01 (rMAG_29)*
acIV (rMAG_690)
Nitrosomonas (rMAG_392)
Chthoniobacteraceae (rMAG_839)
CL500-15 (rMAG_275)*
acI (rMAG_102)
LD12 (rMAG_1314)
Epi.
Hypo.
May
Jun
Jul
Aug
Sep
Oct
Nov
Dec
Jan
Feb
Mar
Apr

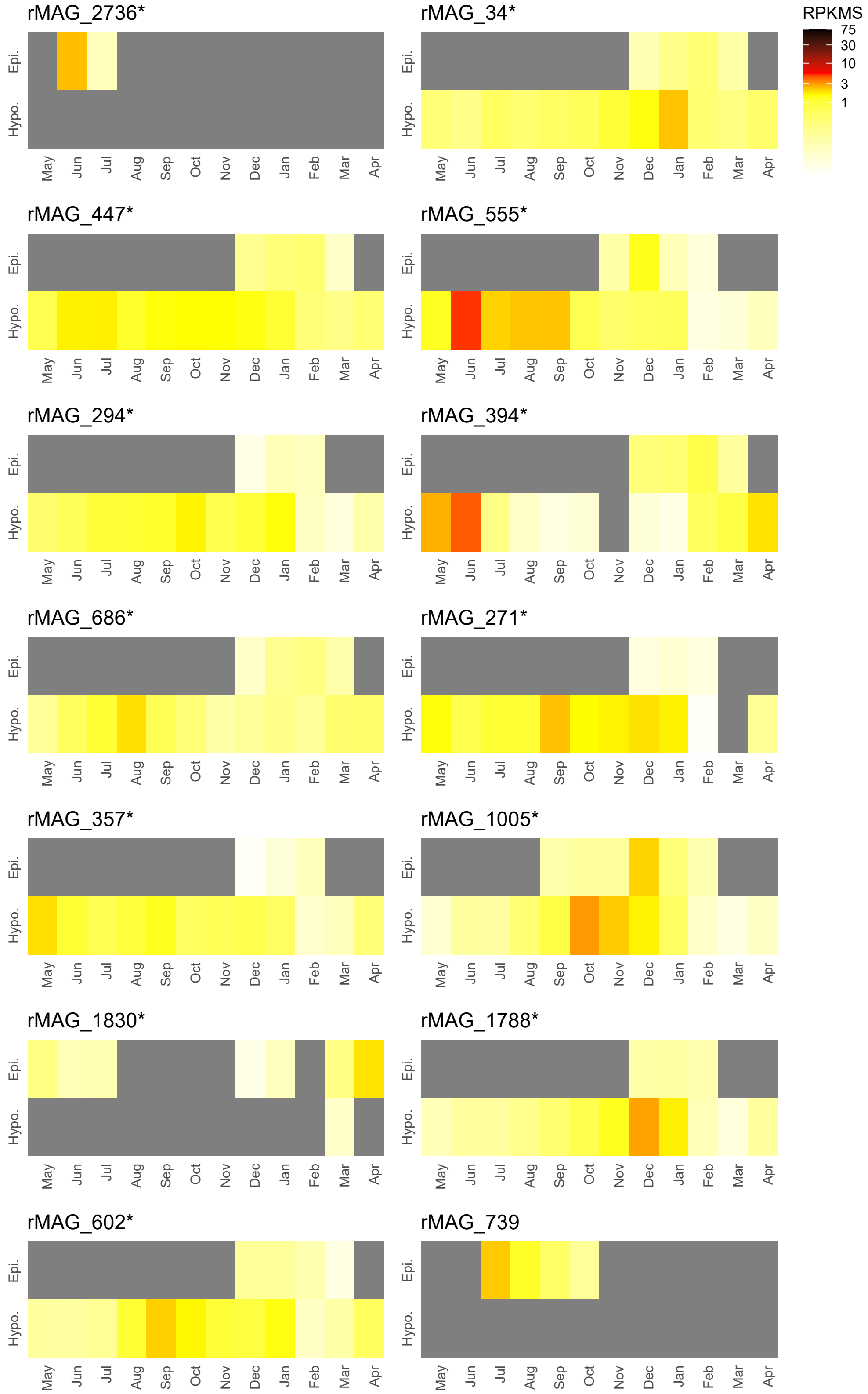

Figure S3. Relative abundances (RPKMS) of representative rMAGs across the 24 samples. Asterisks indicate the top 15 rMAGs with the highest nonsynonymous SNV ratios (delineated in Fig. 3a; n = 15). Gray cells indicate RPKMS = 0 (i.e., not detected). The stratification period was from May to December. Epi., Epilimnion; Hypo., Hypolimnion.
