# Supplementary material for: Long-Read-Resolved, Ecosystem-Wide Exploration of Nucleotide and Structural Microdiversity of Lake Bacterioplankton Genomes
Source: mSystems. 2022 Aug 8;7(4):e00433-22. doi: 10.1128/msystems.00433-22 (PMC9426551; doi:10.1128/msystems.00433-22)
Supplement: FIG S4 [file msystems.00433-22-s0004.pdf]

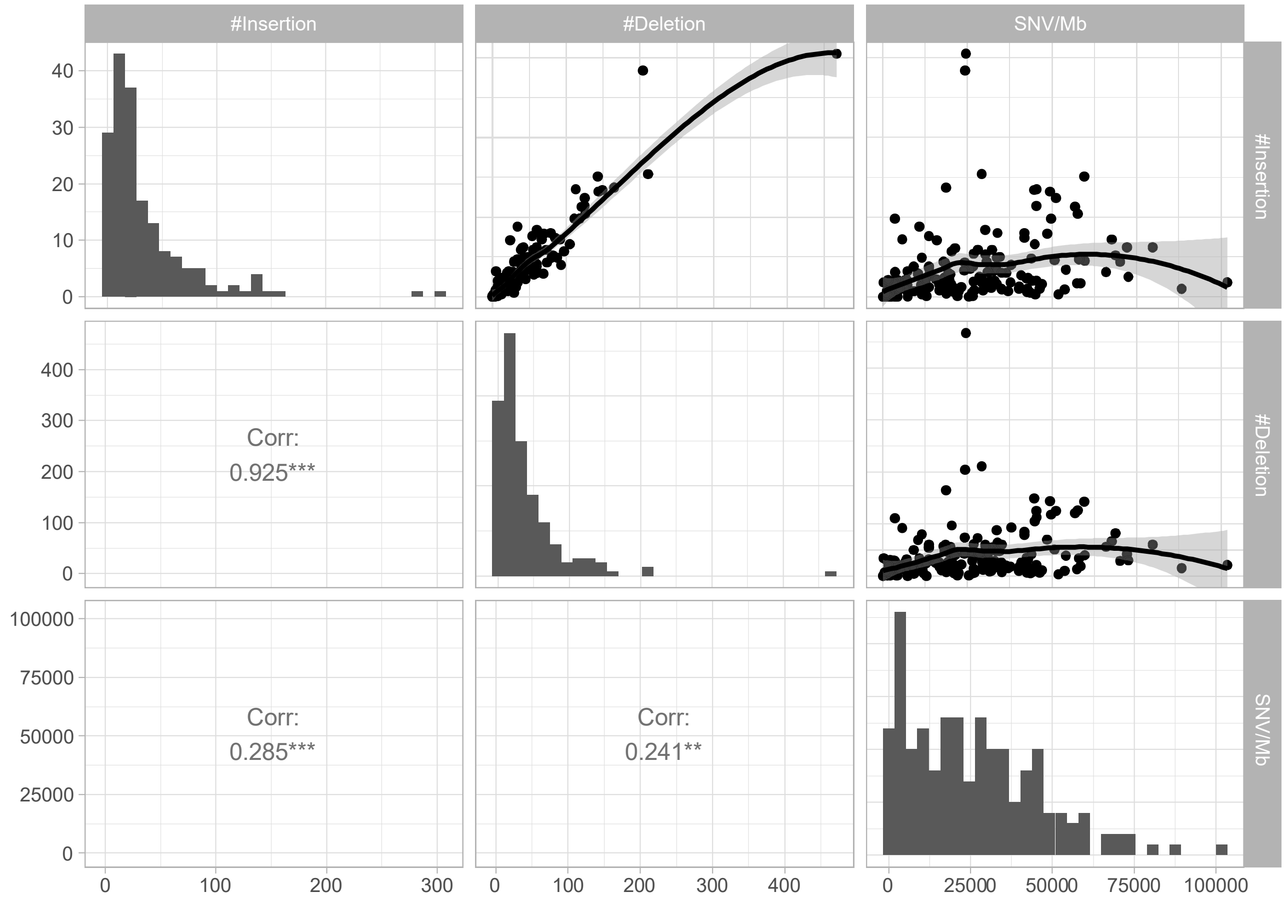

Figure S4. Pairwise plots (upper right panels) among the number of insertions, deletions, and SNVs per 1 Mb. Data were from the representative sample for each rMAG. Solid line represents local regression (loess); 95% confidence intervals are shaded gray. Histograms on diagonal panels indicate the distribution of each parameter. Bottom left panels show the Pearson correlation (r) with *** and ** indicating p-values of < 0.001 and < 0.01, respectively.
