# Supplementary material for: Long-Read-Resolved, Ecosystem-Wide Exploration of Nucleotide and Structural Microdiversity of Lake Bacterioplankton Genomes
Source: mSystems. 2022 Aug 8;7(4):e00433-22. doi: 10.1128/msystems.00433-22 (PMC9426551; doi:10.1128/msystems.00433-22)
Supplement: FIG S5 [file msystems.00433-22-s0005.pdf]

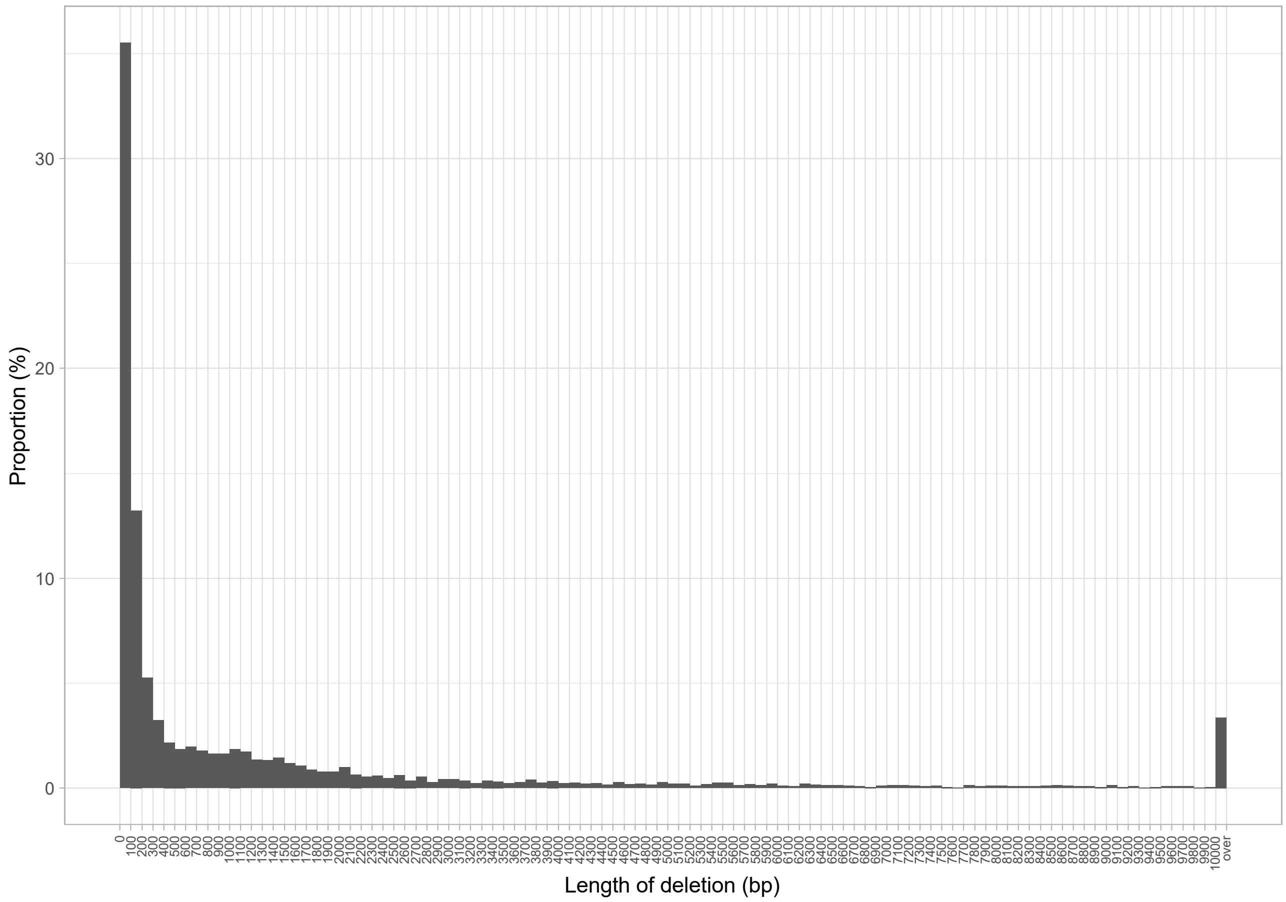

Figure S5. Length distribution of the 9471 deletions detected among the 178 rMAGs. Data were from the representative sample for each rMAG.
