# Supplementary material for: Long-Read-Resolved, Ecosystem-Wide Exploration of Nucleotide and Structural Microdiversity of Lake Bacterioplankton Genomes
Source: mSystems. 2022 Aug 8;7(4):e00433-22. doi: 10.1128/msystems.00433-22 (PMC9426551; doi:10.1128/msystems.00433-22)
Supplement: FIG S6 [file msystems.00433-22-s0006.pdf]

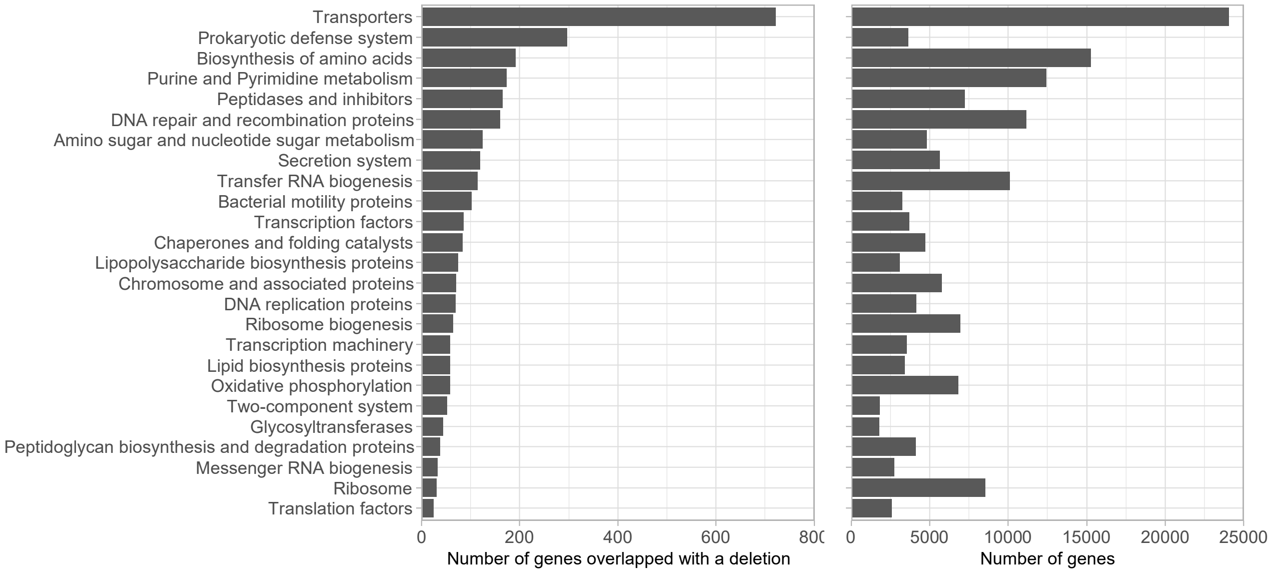

Figure S6. The number of genes in total (right) and those overlapped with a deletion (left) in each gene functional category among the 178 rMAGs analyzed. Data were from the representative sample for each rMAG. Gene categories are sorted by the number of genes overlapped with a deletion.
