# Supplementary material for: Long-Read-Resolved, Ecosystem-Wide Exploration of Nucleotide and Structural Microdiversity of Lake Bacterioplankton Genomes
Source: mSystems. 2022 Aug 8;7(4):e00433-22. doi: 10.1128/msystems.00433-22 (PMC9426551; doi:10.1128/msystems.00433-22)
Supplement: FIG S7 [file msystems.00433-22-s0007.pdf]

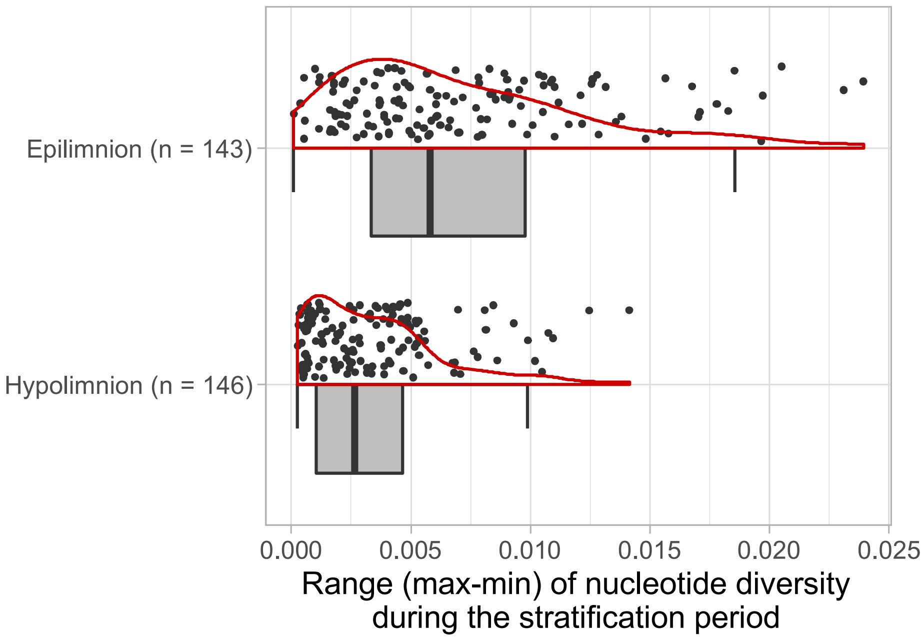

Figure S7. Distribution (half-violin-boxjitter plot) of the range (maximum value–minimum value) of the nucleotide diversity of each rMAG (represented by each point) during the stratification period (May to December). The rMAGs for which the nucleotide diversity could be calculated for more than four out of the eight months in each of the water layers were included in the analysis. The range were significantly broader in the epilimnion than in the hypolimnion, according to the Wilcoxon rank sum test.
