# Supplementary material for: Long-Read-Resolved, Ecosystem-Wide Exploration of Nucleotide and Structural Microdiversity of Lake Bacterioplankton Genomes
Source: mSystems. 2022 Aug 8;7(4):e00433-22. doi: 10.1128/msystems.00433-22 (PMC9426551; doi:10.1128/msystems.00433-22)
Supplement: FIG S8 [file msystems.00433-22-s0008.pdf]

Fig. S8a

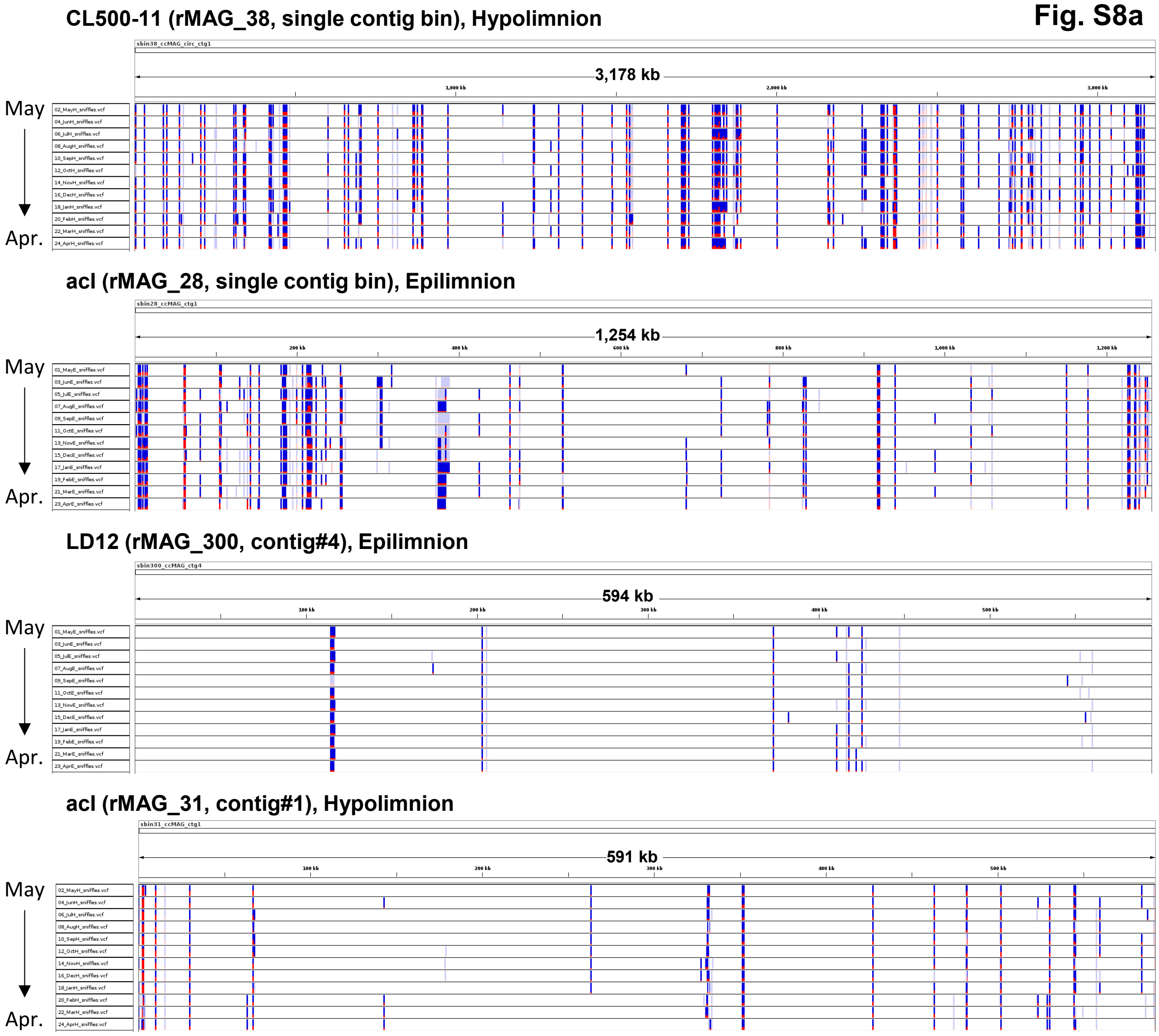
CL500-11 (rMAG_38, single contig bin), Hypolimnion
3,178 kb
May
Apr.
acI (rMAG_28, single contig bin), Epilimnion
1,254 kb
May
Apr.
LD12 (rMAG_300, contig#4), Epilimnion
594 kb
May
Apr.
acI (rMAG_31, contig#1), Hypolimnion
591 kb
May
Apr.

rMAG_354 contig#3 ~700kb, May hypolimnion

Fig. S8b

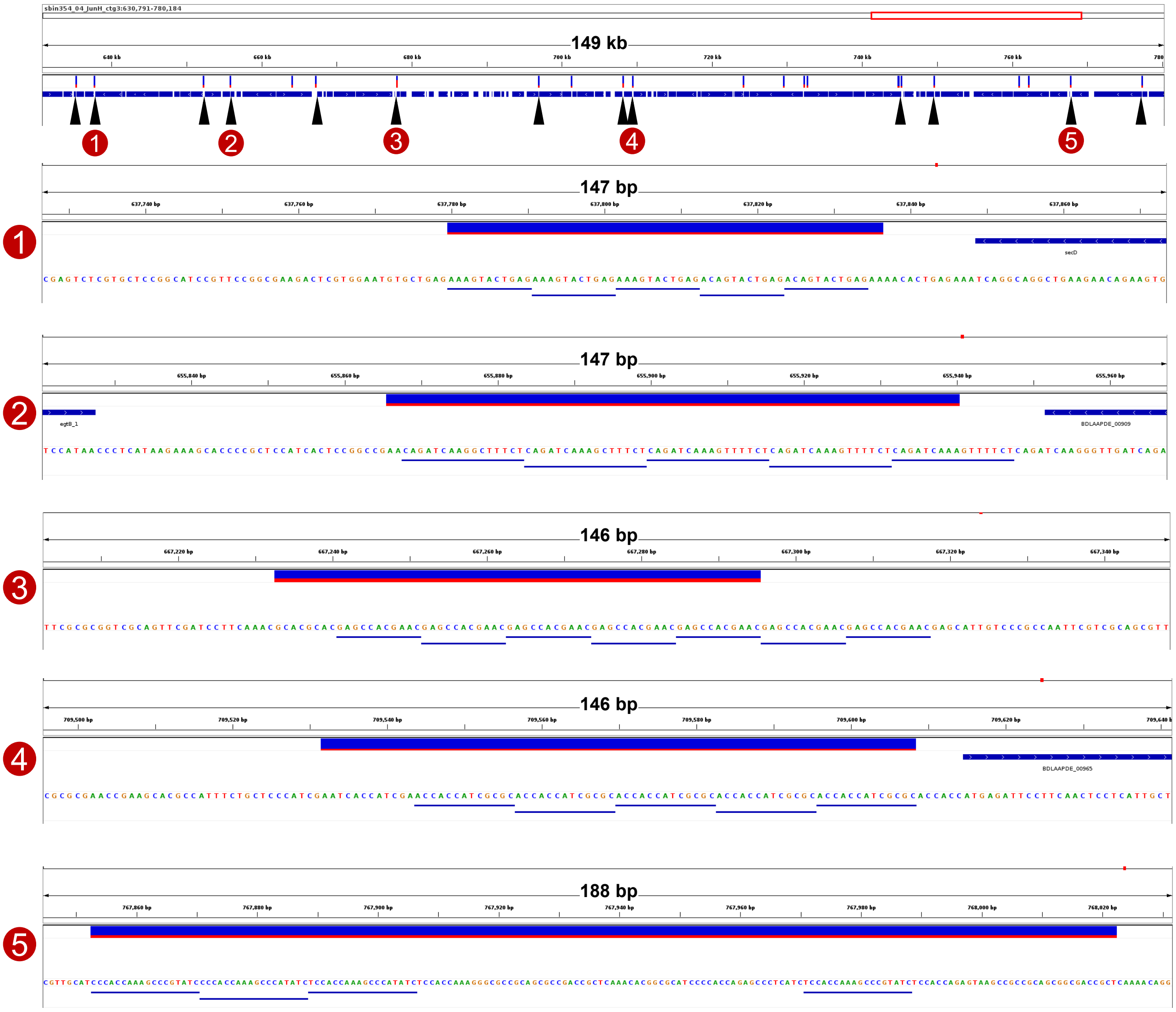

Fig. S8c

rMAG_305 (Rhodoferax), Epilimnion

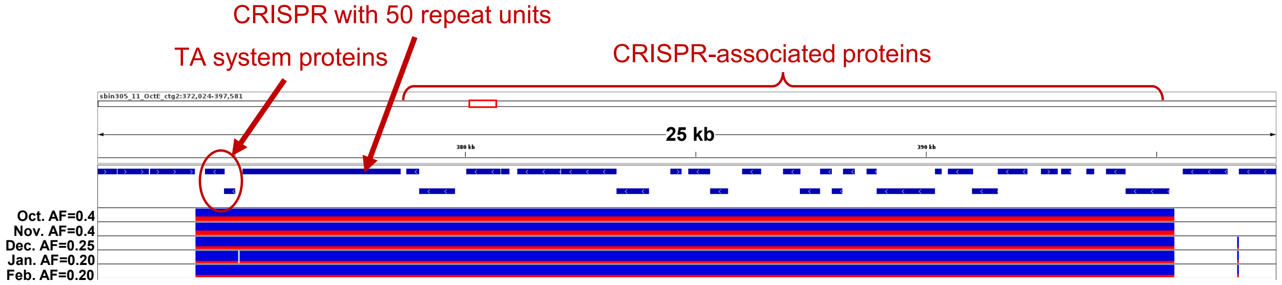

rMAG_1349 (Verrucomicrobiota), Epilimnion

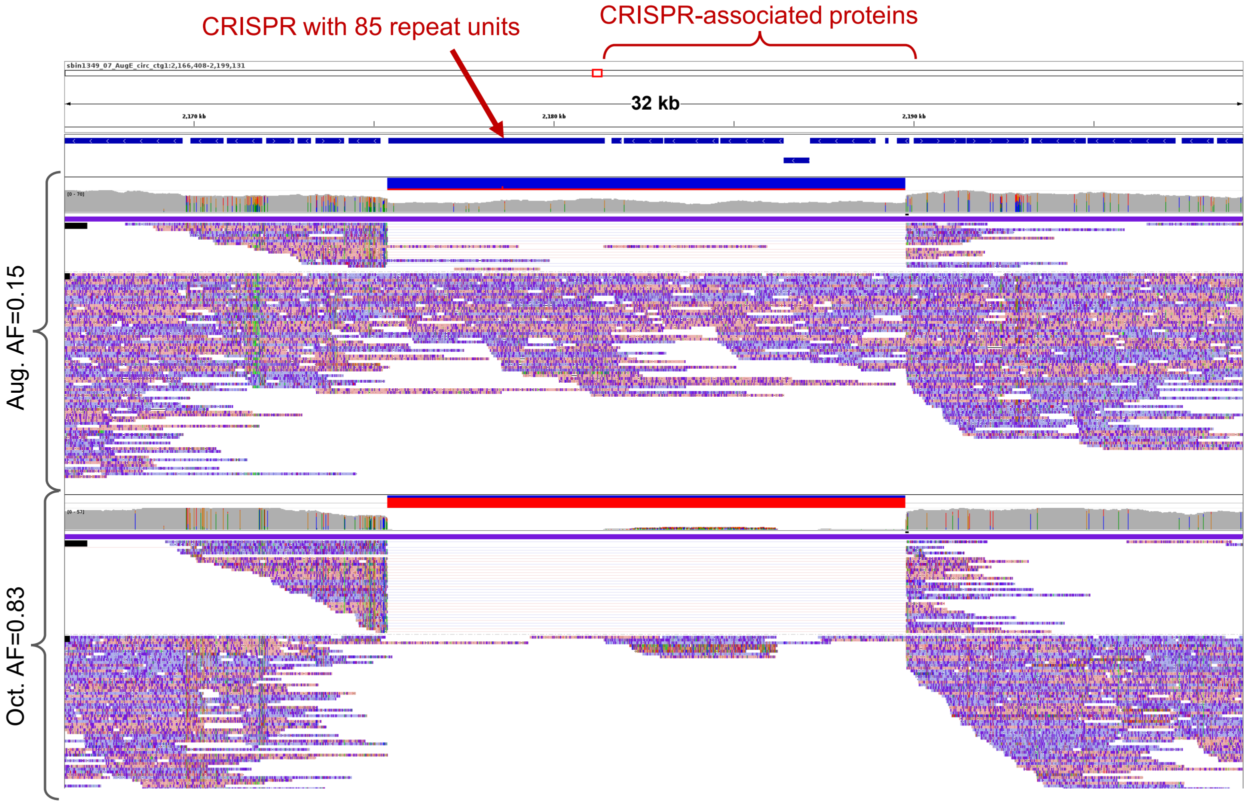

Fig. S8d

rMAG_37 (Verrucomicrobiota), Hypolimnion

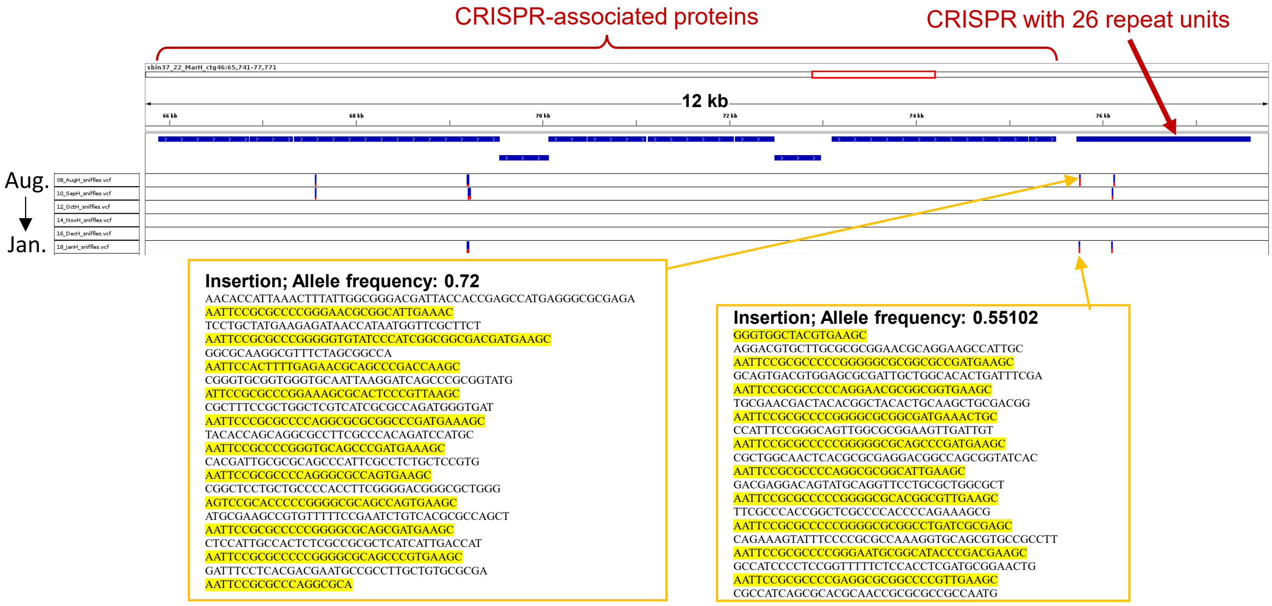

rMAG_271 (Nitrospira), Hypolimnion

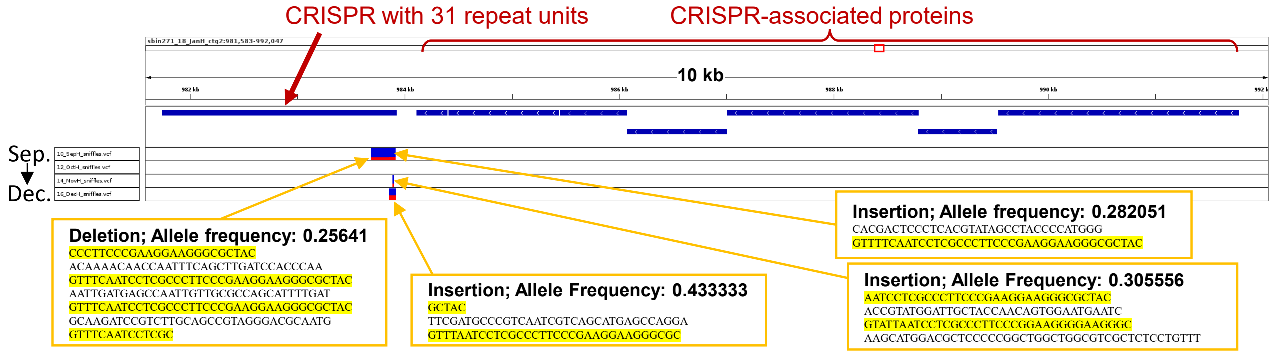

Figure S8. SVs visualized on Integrative Genomics Viewer (IGV). The locations of SVs on an rMAG sequence were indicated by blue and red colored symbols based on the variant call format (.vcf) file generated by Sniffles for each sample. The allele frequency (AF) of each SV was represented as the height of the red-colored part against the blue-colored part. Shaded SVs are those filtered out by Sniffle software due to poor or inconsistent support with read mapping results. (a) Temporal profile of SVs in four representative rMAGs that showed continuously high read coverage during the study period in either of the water layers. The top two rMAGs are single-contig bins while the longest contig was displayed for the other two rMAGs. SV positions in each sequence were chronologically sorted from top (May 2018) to bottom (April 2019). Most SVs were continuously present during the study period. (b) Deletions on intergenic tandem repeats in rMAG_354 (Phycisphaerales of the phylum Planctomycetota). The top panel indicates the SV profile in a representative genomic region of the rMAG. The blue boxes shown below the SVs denotes ORFs. Black arrows indicate intergenic deletions; red circled numbers indicate those involve tandem repeats, for which enlarged visualizations are shown in the bottom panels. Nucleotide sequences and repeat motifs (blue lines below the sequence) are shown in the enlarged panels. (c) Deletions involving a CRISPR–Cas system. In the top case (rMAG_305), the deletion was continuously detected from October to February in the epilimnion, with decreasing allele frequency. The deletion also included TA system proteins downstream of the CRISPR. In the bottom case (rMAG_1349), the allele frequency shifted more quickly from 0.15 in August to 0.83 in October. The coverage track and pileup of the mapped long reads were shown for each sample and indicated that many reads were aligned to the SV region in August while most of the reads bridged the edges of the SV region in October. (d) SVs associated with variation in CRISPR spacer sequences. Both rMAGs showed a shift of SV pattern in the CRISPR sequences during consecutive months in the hypolimnion. SV type, allele frequency, and the sequence involved in each SV are shown in an orange box, in which CRISPR repeat sequences are shaded yellow. Note that sequences shown for insertion were predicted using sequences of the mapped raw long reads and thus unpolished (i.e., error-prone).
